# Canthin-6-One Accelerates Alpha-Synuclein Degradation by Enhancing UPS Activity: Drug Target Identification by CRISPR-Cas9 Whole Genome-Wide Screening Technology

**DOI:** 10.3389/fphar.2019.00016

**Published:** 2019-01-28

**Authors:** Ning-Ning Yuan, Cui-Zan Cai, Ming-Yue Wu, Qi Zhu, HuanXing Su, Min Li, JiaoYan Ren, Jie-Qiong Tan, Jia-Hong Lu

**Affiliations:** ^1^State Key Laboratory of Quality Research in Chinese Medicine, Institute of Chinese Medical Sciences, University of Macau, Macao, China; ^2^Mr. and Mrs. Ko Chi Ming Centre for Parkinson’s Disease Research, School of Chinese Medicine, Hong Kong Baptist University, Hong Kong, China; ^3^School of Food Science and Engineering, South China University of Technology, Guangzhou, China; ^4^Center for Medical Genetics, School of Life Sciences, Central South University, Changsha, China

**Keywords:** canthin-6-one, Parkinson’s disease, alpha-synuclein, ubiquitin-proteasome-system, CRISPR/Cas9, RPN2/PSMD1, PKA

## Abstract

Parkinson’s disease (PD) is the second most common neurodegenerative disorder characterized by the accumulation of protein aggregates (namely Lewy bodies) in dopaminergic neurons in the *substantia nigra* region of the brain. Alpha-synuclein (α-syn) is the major component of Lewy bodies in PD patients, and impairment of the ubiquitin-proteasome system has been linked to its accumulation. In this work, we developed a tetracycline–inducible expression system, with simultaneous induced expression of α-syn-EGFP and a bright red fluorescent protein marker (mCherry) to screen for potential compounds for degrading α-syn. We identified canthin-6-one as an α-syn lowering compound which promoted both wild type and mutants α-syn degradation in an ubiquitin-proteasome-system (UPS) dependent manner. By CRISPR/Cas9 genome-wide screening technology, we identified RPN2/PSMD1, the 26S proteasome non-ATPase regulatory subunit 1, as the targeting gene for pharmacological activity of canthin-6-one. Finally, we showed that canthin-6-one up-regulates PSMD1 and enhances UPS function by activating PKA.

## Introduction

Many neurodegenerative diseases share a common pathogenic mechanism: the aggregation of mis-folded proteins lead to cellular toxicity and proteostatic collapse. In Parkinson’s disease (PD), the aggregation-prone protein alpha-synuclein (α-syn) is both the pathological feature and the causative factor (Stefanis, 2012). Overexpression of wild-type (WT) and mutant α-syn in transgenic mice as well as in transgenic flies caused progressive locomotor defects with dopaminergic neuron loss and intracytoplasmic inclusions (Blesa and Przedborski, 2014). These findings suggest that decreasing the accumulation of α-syn could be a therapeutic strategy for the treatment of PD (Harper et al., 2005; Williams and Paulson, 2008).

Multiple *in vitro* models have been generated to identify compounds affecting α-syn stability in mammalian cells. For example, an inducible PC12/TetOn cells of α-syn expression have been used to evaluate the activity of select compounds on α-syn degradation, assayed with immunoblotting (Batelli et al., 2011; Lu et al., 2012; Chen et al., 2014). However, this method is restricted to a low-throughput capability and the screening process is time consuming, labor intensive and expensive. A fluorescence-based system to monitor protein dynamics has emerged recently (Cretich et al., 2006). In this study, we established a tetracycline–inducible expression system, with a bidirectional tet-responsive promoter that binds the Tet-On 3G transactivator protein in the presence of doxycycline, allowing simultaneous induced expression of α-syn-EGFP and a bright red fluorescent protein marker (mCherry) as the reference. In this system, any compound that selectively affects α-syn stability would change the ratio of EGFP/mCherry, which can be monitored by microplate reader efficiently. The screening identified canthin-6-one as a selective α-syn lowering compound from a pool of about 300 natural compounds. Autophagy-lysosome pathway and ubiquitin-protease system are two major route for α-syn degradation. By using ALP and UPS inhibitor, we confirmed that canthin-6-one induced α-syn degradation dependent of UPS function.

**Figure 1:**
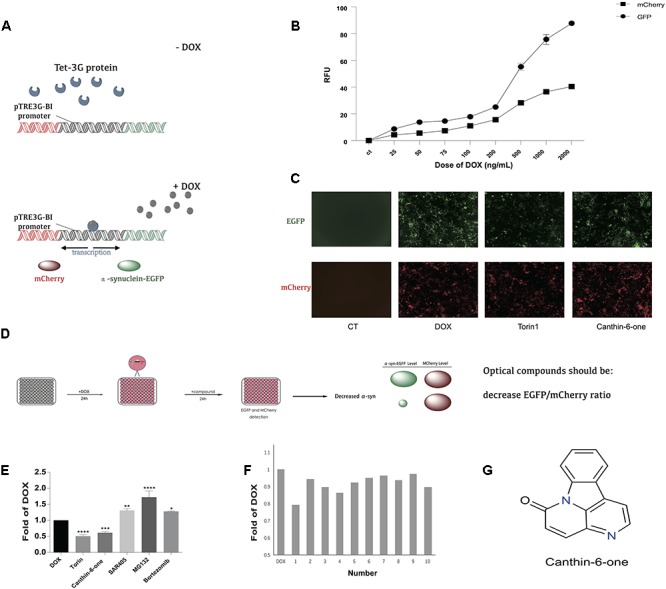
Canthin-6-one was identified as an α-syn lowering compound by using Tet-on 3G α-syn-EGFP/mCherry dual fluorescence system. **(A)** The principle of Tet-on 3G α-syn-EGFP/mCherry dual fluorescence system. **(B)** Induction of α-syn-EGFP (Ex/Em = 488/525 nm) and mCherry (Ex/Em = 587/610 nm) fluorescence signals by adding different concentration of doxycycline. The fluorescence signals were recorded in a plate reader. **(C)** Removing DOX, change of EGFP and mCherry after treating with or without Canthin-6-one in fluorescence microscope, torin1 (0.2 μM) as the positive control. **(D)** The whole flow chart for compound selection. **(E)** Inducible Tet-on 3G α-syn-EGFP/mCherry cells were induced with 0.2 μg/mL DOX for 24 h. Cells were then treated with 15 μM Canthin-6-one, 0.2 μM trion1, 1 μM SAR405, 5 nM Bortezomib and 10 mM MG132 for 24 h after removing DOX. Bar chart shows the microplate reader analysis of EGFP/mCherry ratio. **(F)** The bar chart of potential 10 compounds from 300 according to the EGFP/mCherry ratio by microplate reader analysis. **(G)** The chemical structure of Canthin-6-one. **(F)** ^∗^*P* < 0.05, ^∗∗^*P* < 0.01, ^∗∗∗^*P* < 0.001, and ^∗∗∗∗^*P* < 0.001. Error bars (mean ± SD). One-way ANOVA with Student-Newman-Keuls as *post hoc* tests.

Canthin-6-one, 6H-Indolo[3,2,1-de] [1,5] naphthyridin-6-one, is an indole alkaloid. Its molecular weight is 220.231 g/mol and chemical structure is shown in Figure 1G. Canthin-6-one can be extracted from many plants (Zanthoxylum chiloperone (Ferreira et al., 2002), Aerva lanata (Zapesochnaya et al., 1992), the roots of Eurycoma longifolia (Mitsunaga et al., 1994), Ailanthus altissima (Anderson et al., 1983), and Simaba ferruginea A. St.-Hil. (Gazoni et al., 2018), which has a number of activities, such as against Leishmania (Ferreira et al., 2002), diuretic, anti-inflammatory (Zapesochnaya et al., 1992), antimalarial (Kuo et al., 2003), and antifungal (Gazoni et al., 2018). This is the first time reported canthin-6-one as a UPS activator to lower α-syn.

Identifying the drug targets of pharmacologically effective compounds has long been a challenging task. RNA interference (RNAi) has been the predominant method within the last decade to identify the critic molecules (Berns et al., 2004; Boutros et al., 2004). However, its utility is limited by high off-target effects, incomplete suppression of target genes and time-consuming screening process (Echeverri et al., 2006; Jackson et al., 2006). Recently, the CRISPR-Cas9 library has been demonstrated to be an efficient tool to interrogate gene function on a genome-wide scale (Shalem et al., 2014). Thus we applied a genome-scale CRISPR-Cas9 knockout (GeCKO) library by combining with fluorescence-based flow cytometric sorting to identify potential targets of canthin-6-one. The screening identified a candidate gene PSMD1 gene which is requirement for canthin-6-one-induced α-syn degradation. Consistently, PSMD1 shows an increased expression after canthin-6-one treatment. The expression of PSMD1 is driven by the PKA as PKA inhibitor blocked the canthin-6-one-induced PSMD1 up-regulation as well as the UPS activation. Collectively, we identified Canthin-6-one as an α-syn lowering compound and find PSMD1 as the drug targeting gene by CRISPR-Cas9 whole genome-wide screening technology.

## Materials and Methods

### Generate Stable Cell Line of α-Synucleinopathy

To generate HEK293 double-stable Tet-On 3G mCherry-α-syn-EGFP inducible stable cell line, the first step is to establish the Tet-On 3G stable-expression cell line by G418 selection. Then we cotransfected PTRE-BI-mCherry-α-syn-EGFP plasmid into Tet-On 3G cell line along with a linear selection marker, hygromycin. Tet-On 3G mCherry-α-syn-EGFP inducible stable clones are selected by hygromycin selection.

### Reagents and Antibodies

Canthin-6-one (B30357) was purchased from Ye Yuan company (Shanghai, China), the purity value ≥98%. Anti-α-syn antibody (610786) was purchased from BD Transduction Laboratories. Anti-β-actin (4970), phospho-PKA (5661), anti-PKA (4782) goat anti-mouse (7076), anti-LC3 (2775), and goat anti-rabbit (7074) secondary antibodies were purchased from Cell Signaling Technology. Anti-PSMD1 (SAB2104781-100UL) was purchased from sigma. CQ (C6628) and H-89 (B1427) were purchased from Sigma-Aldrich. SAR405 (A8883), MG-132 proteasome inhibitor (A2585), and Bortezomib (A2614) were purchased from APExBIO. Lipofectamine 3000 reagent (L30000015) was purchased from Invitrogen. A proteasome activity kit was purchased from Promrga. GeCKO library (1000000049) was purchased from addgene. Tet-on-3G bidirectional inducible expression system with mCherry (631338) and linear hygromycin marker (631625) were purchased from CLONTECH.

### Cell Lines and Cell Culture

PC12 cells were grown in DMEM, supplemented with 5% horse serum and 10% FBS. Inducible PC12 cells were grown in DMEM, supplemented with 5% horse serum and 10% FBS containing 200 μg/mL G418 and 150 μg/mL Hygromycin B. HEK293 cells were grown in DMEM, supplemented with 10% FBS. Inducible HEK293 Tet-On 3G mCherry-α-syn-EGFP inducible stable cells were grown in DMEM, supplemented with 10% FBS containing 200 μg/mL G418 and 150 μg/mL Hygromycin B. All cells were grown in a humidified incubator maintained at 37°C with 5% CO_2_.

### Western Blotting Analysis

Cells were lysed with a RIPA lysis buffer (1 mM EDTA, 150 mM NaCl, 50 mM TRIS–HCl, 0.35% sodium deoxycholate, 1% NP40, and 1 mM PMSF), and the protein concentration was determined with a BCA kit. An equal amount of protein was resolved on SDS–PAGE gel, and then transferred onto PVDF membrane. Membranes were blocked in 5% non-fat milk at room temperature for 2 h, and then probed with the primary antibody and the HRP-conjugated secondary antibody. Bands were visualized using an ECL kit.

### Proteasome-GloTM Cell-Based Assays

The chymotrypsin-like protease activity was determined by the Proteasome-Glo^TM^ activity kit according to the manufacturer’s mannual. Briefly, 8000 cells/well were added to a 96-well plate and treated with 15 μM canthin-6-one for 24 h. Prepare the Proteasome-GloTM Reagent by adding the Proteasome-GloTM Substrate to the reconstituted Luciferin Detection Reagent. Allow the reagent to equilibrate to room temperature. Remove the 96-well plate containing cells from the incubator, and allow the plate to equilibrate to room temperature. Add 100 μL of Proteasome-GloTM Cell-Based Reagent to each 100 μL of sample and appropriate controls as needed. Cover the plate using a plate sealer or lid. Mix the contents of the wells at 700 rpm using a plate shaker for 2 min. Incubate at room temperature for a minimum of 10 min. Measure the luminescence of each sample in a plate-reading luminometer as directed by the luminometer manufacturer.

### Plasmids and Transfection

Ub-G76V-GFP and Ub-R-GFP were purchased from addgene. Cells were transfected with plasmids using lipofectamine 3000 (invitrogen L3000008) according to the manufacturer’s protocol.

### CRISPR-Cas9 Whole Genome-Wide Screening

#### Lentivirus Production and GeCKO Library Purification

Lentivirus was produced as previously described by Dr. Feng Zhang (Shalem et al., 2014). Briefly, 4 μg of a lentiCRISPR plasmid library (#1000000048, Addgene) was co-transfected with 2 μg of pVSVg (#8454, Addgene), and 6 μg of psPAX2 (#12260, Addgene) packing plasmids into HEK293T cells in a 10 cm^2^-dish using Lipofectamine 2000 (Invitrogen) follow the manufacturer’s protocol. After 48 h, the media was collected and centrifuged at 3,000 rpm at 4 °C for 10 min to pellet cell debris. The supernatant was filtered through a 0.45 μm filter (Pall, KA2DBLP6G). The virus was ultracentrifuged (Sorvall) at 24,000 rpm for 2h at 4°C and then resuspended overnight at 4°C in D10 supplemented with 1% BSA.

#### CRISPR-Cas9-Mediated Whole Genome Screening

Cells were transduced with the GeCKO library at a MOI of 0.1–0.3 aiming to ensure that most cells received only 1 viral construct. Briefly, 2 × 10^7^ cells per 10 cm dish were plated in DMEM medium containing 10% fetal calf serum. Lentivirus was added to each dish with 8 μg/mL polybrene (Sigma). 48 h after infection, the culture media was replaced with fresh media containing 1 μg/mL puromycin. 96 h after puromycin selection, 10% cells with high level of GFP signal after Canthin-6-one treatment were collected for genomic DNA isolation.

#### Genomic DNA Sequencing and Data Analysis

PCR was performed to amplify the sgRNA region and the PCR products were sequenced using a HiSeq 2500 (Illumina) as described by Dr. Feng Zhang. Forward primer: CTTGTGGAAAGGACGAAACA, Reverse primer: GCCAATTCCCACTCCTTTCA. Raw FASTQ files were demultiplexed using the FASTX-Toolkit^1^. Then reads were aligned to the index using the Bowtie aligner. The numbers of reads for each unique sgRNA for a given sample were normalized after alignment.

### Real-Time qPCR

The results of quantitative PCR was analyzed by 2^-ΔΔCT^ method to calculate the fold change in the levels of PSMD1, PSMA1, PSMA6, and PSMA7. The forward and reverse sequences of the PCR primers are list in the Table 1.

**Table 1:** qRT-PCR primers.

Genes of interest	Forward	Reverse
PSMD1	GGGGCTTTTGAGGAGTCTCT	GCAAATCTGCATTTTCCACA
PSMA6	ATTACCATTTTTTCTCCCG	GCTGTCATTCCTGTCATCAC
PSMA1	TGTTTGACAGACCACTTCCT	TCTTCAAGACCATCCAGGAA
PSMA7	AACGTCTGTATGGCCTTTGC	GTCACTGGGTCCTCCACTGT
β-actin	AGTGTGACGTTGACATCCGT	TGCTAGGAGCCAGAGCAGTA

### Statistical Analysis

Each experiment was performed at least 3 times, and the results were presented as mean ± SD. The difference was analyzed by one-way analysis of variance (ANOVA) followed by the Student-Newman-Keuls test using the SigmaPlot 11.0 software packages. A probability value less than 0.05 was considered to be statistically significant.

## Results

### Canthin-6-One Was Identified as an α-syn Lowering Compound by Using Tet-on 3G α-syn-EGFP/mCherry Dual Fluorescence System

To identify compounds which can degrade α-syn levels in human cells, we engineered a plasmid encoding human α-syn fused with monomeric green fluorescent protein (α-syn-EGFP). To distinguish modifiers that regulate α-syn protein levels from those that regulate transgene transcription, we cloned α-syn-EGFP into the vector pTRE3G-BI-mCherry, which contains a bidirectional doxycycline (DOX)-inducible promoter. After establishing the Tet-On 3G stable-expression cell line by G418 selection, we cotransfected established PTRE-BI-mCherry-α-syn-EGFP plasmid into Tet-On 3G cell line along with a linear selection marker, hygromycin. Double-stable Tet-On 3G mCherry-α-syn-EGFP inducible stable clone were selected by hygromycin. DOX addition induces a conformational change in the transactivator, resulting in promoter binding and gene expression, which allows the simultaneous, equivalent, co-induced expression of a green fluorescent protein (α-syn-EGFP) and a red fluorescent protein marker (mCherry) (Figure 1A). Then Tet-on 3G α-syn-EGFP/mCherry cells were further analyzed to determine the best DOX concentration. After adding 0.025, 0.05, 0.075, 0.1, 0.2, 0.5, 1, and 2 μg/mL dox, then remove dox for 24 h, microplate reader analysis of both EGFP (Ex/Em = 488/525 nm) and mCherry (Ex/Em = 587/610 nm) demonstrated that administration of DOX at a concentration of 0.2 μg/mL was sufficient to obtain maximal stable EGFP/mCherry ratio (Figure 1B). The EGFP-to-mCherry fluorescence ratio serves as a read-out for identifying compounds that directly influence the level of α-syn while using high-throughput Microplate reader.

α-syn can be degraded by both autophagy and ubiquitin-proteasome system (UPS) (Webb et al., 2003). We used the inhibitors/inducers of autophagy and UPS to verify our established Tet-on 3G tetracycline-inducible co-expression system. Briefly, cells were incubated with DOX for 24 h to induce the expression of α-syn-EGFP and mCherry, and then the DOX was removed to stop the gene expression. Cells then were incubated in medium containing either autophagy inhibitor 1.5 μM SAR405 (APExBIO), or proteasome inhibitor 5 nM Bortezomib (APExBIO) and 10 μM MG132 (APExBIO). Both inhibitors led to a slight increase of α-syn levels in the cell lines. Besides, we also used autophagy inducer 0.1 μM Torin 1 (LC laboratory), a stimulator of autophagy known to degrade α-syn. Torin 1 promoted the clearance of α-syn as expected (Figure1E). These results demonstrated that the EGFP/mCherry ratio is able to reflect α-syn levels in cells, and thus can be used to screen for compounds that decrease α-syn levels in cells.

**Figure 2:**
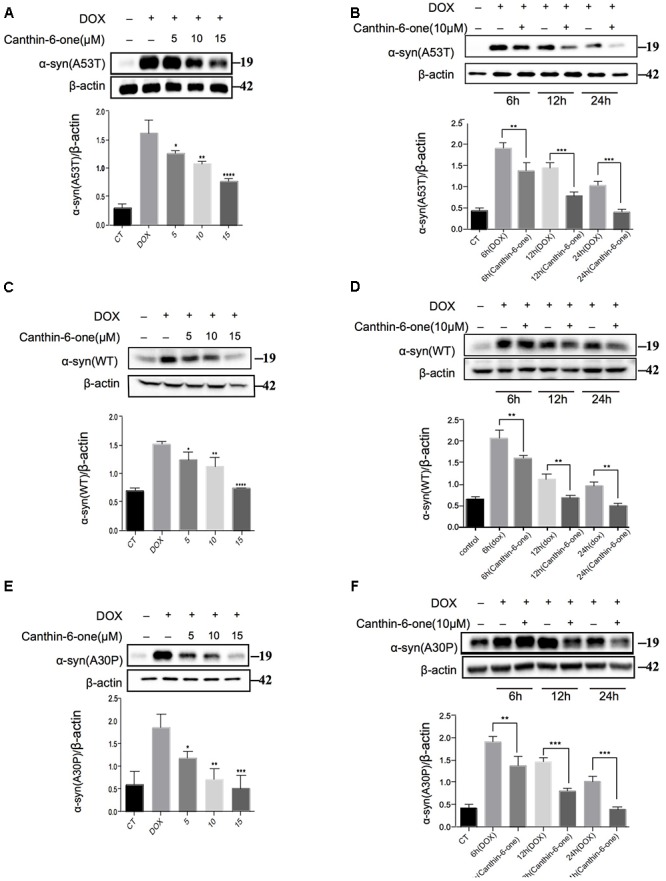
Canthin-6-one promotes degradation of wild type and mutant α-syn in inducible PC12 cell models. Cells were incubated with 2 μg/mL DOX for 24 h, then the DOX was removed and cells were treated with 5, 10, and 15 μM of Canthin-6-one for 24 h. Cell lysates were subjected to western blotting analysis. Canthin-6-one degrades WT, A53T, and A30P α-syn in PC12 inducible cells in both dose-dependent **(A–E)** and time-dependent manner **(B–F)**. ^∗^*P* < 0.05, ^∗∗^*P* < 0.01, ^∗∗∗^*P* < 0.001, and ^∗∗∗∗^*P* < 0.001. Error bars (mean ± SD). One-way ANOVA with Student-Newman-Keuls as *post hoc* tests.

Using the duel-report system, we screened a total number of 300 compounds for the activities of decreasing α-syn. In the primary screening, we identified 10 compounds which lowered the α-syn-EGFP/mCherry ratio at the concentration of 5 μM when treated for 24 h (Figure 1F) (Supplementary Table S1). Then, we performed the MTT assay to evaluate the toxicity of Canthin-6-one and found that Canthin-6-one did not affect cell viability until 20 μM (Supplementary Figure S1A). In the second round validation, we tested the effects of compounds on the α-syn tet-on inducible PC12 cells which express α-syn after induction with the DOX (Greene and Tischler, 1976). One hit that satisfied our criteria was Canthin-6-one. Canthin-6-one decreased GFP-to- mCherry fluorescence ratio in HEK293 tet-on cells and also resulted in a decrease of WT, A30P, and A53T α-syn in PC12 inducible cells in both time and dose dependent manners (Figure 2). These results indicated that Canthin-6-one is a potent α-syn-lowering compound (Figure 1C,D, 2). Besides, cellular co-localization of phosphorylated tau and α-synuclein has been observed in sporadic PD (Arima et al., 1999). It is interesting that Canthin-6-one can also decrease phosphorylated tau (AT8) in a SH-SY5Y cell line constantly expressing P301S tau (Supplementary Figure S1B).

### Canthin-6-One Promotes the Degradation of α-syn via Activating UPS

**Figure 3:**
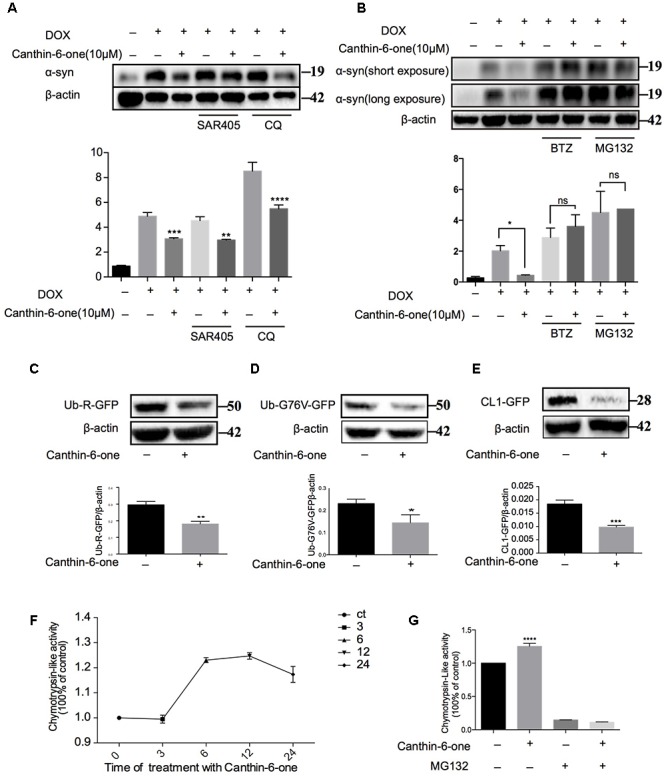
Canthin-6-one promotes the degradation of α-syn via activating UPS. **(A)** Inducible PC12 A53T cells were induced with 2 μg/mL of DOX for 24 h. The cells were then treated with 10 μM of Canthin-6-one, in the presence or absence of autophagy inhibitors SAR405 (1 μM) and CQ (30 μM) for 24 h. Cell lysates were subjected to western blotting analysis. **(B)** Inducible PC12 A53T cells were induced with 2 μg/mL DOX for 24 h. The cells were then treated with 10 μM Canthin-6-one, in the presence or absence of proteasome inhibitors MG132 (0.5 μM) and BTZ (1 μM) for 24 h. Cell lysates were then subjected to western blotting analysis. To determine the UPS activity, N2a cells were transfected with Ub-R-GFP (**C**), UbG76V-GFP **(D)**, and then treated with 10 μM Canthin-6-one for 24 h. The cell lysates were subjected to western blotting analysis. **(E)** GFP-CL1 stably expressing SH-SY5Y cells were treated with Canthin-6-one for 24h and the GFP-CL1 level was determined by western blotting. **(F)** The PC12 cells were incubated with or without Canthin-6-one and the chymotrypsin-like peptidase activity was measured by Proteasome-Glo^TM^ Cell-Based Assays kit. **(G)** The effect of Canthin-6-one on chymotrypsin-like peptidase activity can be blocked by co-treatment with MG132. ^∗^*P* < 0.05, ^∗∗^*P* < 0.01, ^∗∗∗^*P* < 0.001, and ^∗∗∗∗^*P* < 0.001. Error bars (mean ± SD). One-way ANOVA with Student-Newman-Keuls as *post hoc* tests.

To figure out the pathway by which Canthin-6-one promoted α-syn degradation, we examined effect of Canthin-6-one on the two major pathways for α-syn degradation, namely, autophagy-lysosome pathway (ALP) and UPS. By applying autophagy and UPS inhibitors, we revealed that Canthin-6-one-induced α-syn degradation can be blocked by proteasome inhibitor either MG132 or Bortezomib, but not by the autophagy inhibitors (Figure 3A,B). The observations suggest that Canthin-6-one promotes α-syn degradation in a UPS-dependent manner and autophagy-independent manner.

Then, we examined the effect of Canthin-6-one on the UPS activity by using three reporters: CL1-GFP, Ub-G76V-GFP, and Ub-R-GFP which are known to be degraded by proteasome (Dantuma et al., 2000). SH-SY5Y cells stably overexpressing CL1-GFP were treated with Canthin-6-one for 24 h and a dramatic degradation of CL1-GFP were observed compared with untreated group (Figure 3E). Ub-76v-GFP and Ub-R-GFP were transfected into N2a cells for 24 h and then treated with or without Canthin-6-one for another 24 h in the presence of CHX (stop protein synthesis). Western blot results showed that Canthin-6-one treatment could efficiently degrade Ub-G76V-GFP and Ub-R-GFP level compared with control group (Figure 3C,D).

Proteins are digested within the core 20S proteasome which is a hollow cylindrical particle containing three types of peptidase activities: chymotrypsin-like, trypsin- like, and caspase-like. The chymotrypsin-like protease activity is often considered the most important in protein breakdown, so we performed experiments to evaluate the cellular proteasome activity with Suc-LLVY-AMC probe (chymotrypsin-like activity). Canthin-6-one treatment promoted the hydrolysis of the specific substrate of the proteasome’s chymotrypsin-like site, suc-LLVY-amc, by 40% in PC12 cell extracts (Figure 3F), and this effect can also be blocked by MG132 (ups inhibitor) (Figure 3G). Taken together, our results showed that Canthin-6-one can activate proteasome activity.

### CRISPR-Cas9 Whole Genome-Wide Screening Identified Several Proteasome Subunit Genes Required for Canthin-6-one-Induced α-syn Degradation

**Figure 4:**
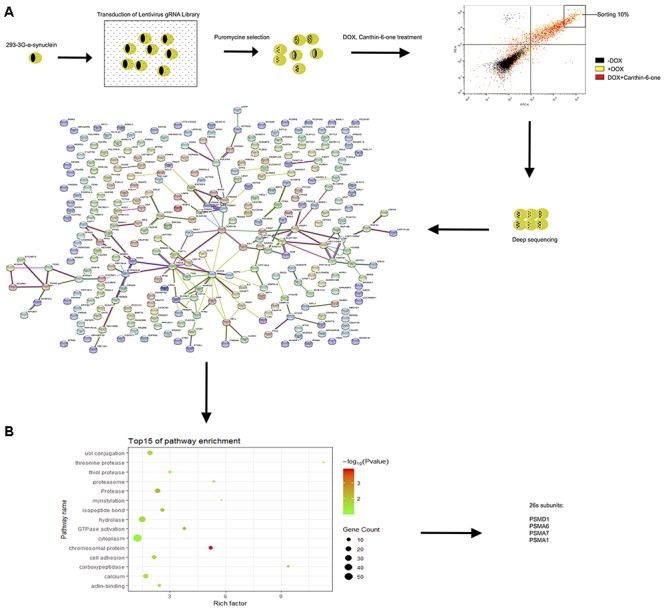
CRISPR-Cas9 whole genome-wide screening identified several proteasome subunit genes required for canthin-6-one-induced α-syn degradation. **(A)** Schematic of the CRISPR/Cas9 screen in Tet-on pTRE3G-BI-mCherry- α-syn -EGFP HEK-293 cells to identify genes required for effecting of canthin-6-one. Cells were transduced with the GeCKO sgRNA library and cells that were successfully transduced were selected with puromycin. After selection, the population was treated with DOX for 24 h, and then removed for canthin-6-one 24-h treatment. Collected the upper right corner 10% of EGFP) by flow cytometry and extracted the genomic DNA for deep sequencing and enriched sgRNAs were identified by bio-information analysis. Interactions of the top 300 candidate genes whose loss alleviate Canthin-6-one efficiency on degradation of α-syn from the STRING database. The nodes represent proteins and the thickness of the connecting lines indicates the confidence level (0.6–0.9). **(B)** Gene Ontology analysis based on their biological process.

To identify the targeting gene of Canthin-6-one for promoting UPS activity, we applied the GeCKO library (version 2.0) that contains 122,417 unique sgRNAs to target the whole genome and has been demonstrated to have good performance in genome-scale screening (Shalem et al., 2014). Tet-on pTRE3G-BI-mCherry-α-syn-EGFP HEK-293 cells were infected with the CRISPR-Cas9 library at 0.3 multiplicity of infection (MOI) to guarantee only one transgene copy number infected in most of the cells. The infected cells were then selected by puromycin for 3 days to remove non-infected cells. Cells infected with GeCKO library were treated with 0.2 μg/mL DOX to induce the expression of EGFP-α-syn for 24 h, and then treated with vehicle or 15 μM Canthin-6-one for another 24 h without DOX. It is reasonable to predict that in cells which the key molecules required for Canthin-6-one -induced α-syn clearance is knocked out, the EGFP-α-syn signal will not decrease after Canthin-6-one treatment. We collected those cells showing higher level of GFP signal after Canthin-6-one treatment (the cell population in the upper right corner 10% of EGFP) by flow cytometry and extracted the genomic DNA for deep sequencing (Figure 4).

**Figure 5:**
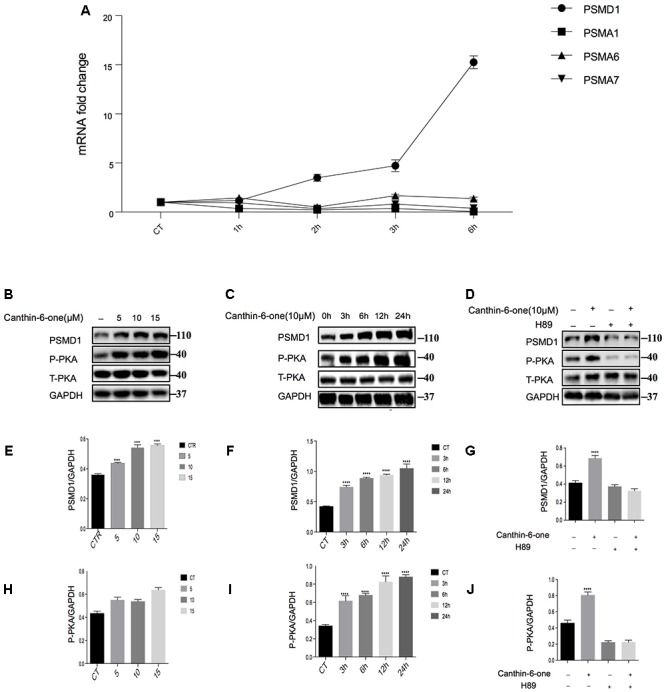
Canthin-6-One promotes PSMD1 expression and enhances proteasome activity and through PKA activation. **(A)** PC12 cells were incubated with 15 μM of Canthin-6-one for 1, 3, and 6 h. Cell lysates were subjected to qRT-PCR analysis. Canthin-6-one promoted PSMD1 expression at mRNA level. **(B,C,E,F)** Canthin-6-one promoted PSMD1 expression in both dose- **(B,E)** and time-dependent manner **(C,F)**. **(B,C,H,I)** Canthin-6-one also promoted PKA phosphorylation in both dose- **(B,H)** and time-dependent manner **(C,I)**. **(D,G,J)** Co- treatment with PKA inhibitor H89 prevented the Canthin-6-one-induced PKA phosphorylation and PSMD1 up-regulation. ^∗^*P* < 0.05, ^∗∗^*P* < 0.01, ^∗∗∗^*P* < 0.001, and ^∗∗∗∗^*P* < 0.001. Error bars (mean ± SD). One-way ANOVA with Student-Newman-Keuls as *post hoc* tests.

After bioinformatics analysis of the sgRNA identity and abundance compared with those cells without Canthin-6-one treatment, we identified four candidate genes which were the proteasome subunits: PSMA1, PSMA6, PSMA7, and PSMD1 (Figure 4). The expression levels of the four genes after Canthin-6-one treatment were determined in PC12 cells by RT-PCR and western blot. As the results showed, the expression of PSMD1 mRNA level was increased to 10-fold after Canthin-6-one treatment, but other subunits did not change (Figure 5A). Consistently, Canthin-6-one also significantly promoted PSMD1 protein expression in both dose- and time-dependent manner (Figure 5B,C,E,F). These results demonstrate that PSMD1 was identified as the potential targeting gene of Canthin-6-one.

### Canthin-6-One Promotes PSMD1 Expression and Enhances Proteasome Activity and Through PKA Activation

Previous studies have revealed that activated PKA promotes the breakdown of short-lived proteins through the UPS (Lokireddy et al., 2015). In the candidate gene list, we found a catalytic subunit of PKA namely PRKACG (Protein Kinase CAMP-Activated Catalytic Subunit Gamma), so we tested whether Canthin-6-one promoted PSMD1 protein expression via activating PKA. The results showed that after treatment of Canthin-6-one in PC12 cells, PKA phosphorylation at Thr197 was dramatically induced in a dose- and time-dependent manner (Figure 5B,C,H,I). To confirm the pathway of Canthin-6-one-induced PSMD1 up-regulation, we used H89, a PKA inhibitor, to inhibit the PKA activation when treated with Canthin-6-one. The results showed that Canthin-6-one-induced PKA phosphorylation was completely blocked by H89, as well as the Canthin-6-one-induced PSMD1 up-regulation (Figure 5D,G,J). The data indicates that Canthin-6-one promotes the UPS activity and α-syn clearance through PSMD1 up-regulation by activating PKA. The raw data has been shown in Supplementary Data Sheet S1.

## Discussion

Parkinson’s disease is a neurodegenerative disorder that is pathologically characterized by the loss of dopaminergic neurons and the presence of intracytoplasmic Lewy bodies (Chung et al., 2001). Mutations in α-syn (A53T, A30P) cause familial PD (Polymeropoulos et al., 1997). Both wild-type WT and mutant forms of α-syn (A53T and A30P) proteins are found in Lewy bodies (Conway et al., 1998). Neuronal overexpression of human α-syn in mice models resulted in progressive accumulation of α-syn, dopaminergic loss and inclusion body formation (Masliah et al., 2000). Transgenic drosophila overexpressing the *SNCA* gene (coding α-syn) also develop PD like pathological and behavior change (Kuo et al., 2010). These similar pathological characteristics between α-syn models and PD suggest that α-syn may play a key role in the pathogenesis of PD and could be the primary therapeutic target for PD. To support this noting, several studies have shown that reducing α-syn levels or inhibiting its aggregation can rescue neurodegeneration in multiple α-synucleinopathy models (Lu et al., 2011; Ardah et al., 2015). By targeting the pathological feature of PD – the accumulation of α-syn, we established a high-throughput cellular model to monitor the degradation of α-syn and identified canthin-6-one as an α-syn lowering compound which also effectively promoted the degradation of three forms of α-syn (WT, A53T, and A30P) in PC12 cells.

α-syn, ubiquitin, as well as several proteasomal subunits can be observed in the Lewy bodies (Betarbet et al., 2005), indicating the direct connection between α-syn and UPS in the pathology of PD. The connection between UPS dysfunction and the pathogenesis of PD is further strengthened by the fact that conditional knockout of PSMD1, the critic subunit of 26S proteasome, resulted in α-syn accumulation phenotype in mice. Overexpression of WT and mutant α-syn *in vitro* led to inhibition of chymotrypsin-like proteasome activity and the composition change of the proteasome (Stefanis et al., 2001). WT and mutant α-syn, particular in their soluble oligomeric and aggregated conformations, inhibit the activity of 26S and 20S proteasome (Emmanouilidou et al., 2010). α-syn direct binding to the β5 subunit of the 20S proteasome or Rpt5 subunit of the 19S proteasome for proteasome inhibition effects (Zhang et al., 2008). It was also reported that a new class of molecules identified to enhance 20S proteolytic activity induced the selective degradation of disordered proteins, α-syn and over structured proteins *in vitro* (Jones et al., 2017). Collectively, these findings revealed the therapeutic potential of UPS activation as a new strategy to accelerate the degradation of α-syn for PD treatment. Though autophagy activation has been well known to promote the α-syn degradation (He et al., 2013;Wang et al., 2016), we applied autophagy and UPS inhibitors in our study and revealed that canthin-6-one promoted the α-syn degradation in a UPS-dependent manner and autophagy-independent manner. By using CL1-GFP reporter system and proteasome activity assay, we confirmed that canthin-6-one is a potent UPS enhancer.

Drug target identification has long been a challenging task. The traditional approaches for drug target identification includes proteomics, microarray and affinity purification. The proteomics and microarray can only provide the information on the changed expression levels of protein and mRNA, which cannot reveal the direct causative connection between drug treatment and changed protein/gene expression. While the affinity purification requires a strong binding and frequently lead to false positive results due to unspecific binding. Recently, the CRISPR-Cas9 system combined with whole genome sgRNA library has been successfully used to identify genes required for a specific phenotype (Joung et al., 2017). The advantage of this approach is unbiased, fast and providing direct causative connection. In the study, we applied the GeCKO library combining with flow cytometry sorting to identify the gene required for Canthin-6-one activity. After GeCKO library lentivirus infection and Canthin-6-one treatment, the top 10% cells showing highest level of GFP signal were collected for deep sequencing. Because loss of genes required for Canthin-6-one-induced α-syn-GFP degradation will results in accumulation of α-syn-GFP. After deep sequencing and bioinformatics analysis of the sgRNA identity and abundance compared with those cells without Canthin-6-one treatment, we identified a candidate gene PSMD1 which encodes a subunit of proteasome. Interestingly, the mRNA and protein levels of PSMD1 dramatically increased after Canthin-6-one treatment by western blot and Q-PCR analysis. We further revealed that Canthin-6-one enhanced PKA phosphorylation at Thr197, a critic event required for PSMD1 up-regulation and UPS activation. The data indicates that Canthin-6-one promotes the UPS activity and α-syn clearance through PSMD1 up-regulation by activating PKA. In this study, CRISPR-Cas9 system proved to be a high efficient, low cost and creative screening approach to identify candidate drug targets. This is also the first attempt to apply the CRISPR/Cas9 genome-wide screening technology to identify the drug targets of the UPS enhancer.

To sum up, our study identified canthin-6-one as a potent UPS activity enhancer, which promoted α-syn degradation. By using CRISPR/Cas9 genome-wide screening technology, the PSMD1 was identified as the targeting gene of canthin-6-one. The findings revealed the therapeutic potential of canthin-6-one in the PD treatment and verified the application of CRISPR/Cas9 genome-wide screening technology for the drug target identification.

## Author Contributions

J-HL designed the experiment and initiated the project. N-NY cloned the gene and established the cell line. C-ZC selected the compound. M-YW and QZ performed the statistical analysis. J-QT contributed to the CRISPR library establishment, bioinformatics, and deep sequencing analysis. N-NY was responsible for qRT-PCR and all Western-blot data. N-NY wrote the first draft of the manuscript. HS, ML and JR wrote the sections of the manuscript. All the authors contributed to manuscript revision, read, and approved the submitted version.

## Conflict of Interest Statement

The authors declare that the research was conducted in the absence of any commercial or financial relationships that could be construed as a potential conflict of interest. The reviewer, JG, declared a shared affiliation at the time of review, though no collaboration, with one of the authors, J-QT, to the handling Editor.

## Footnotes

1 http://hannonlab.cshl.edu/fastx_toolkit/

## Supplementary Material

FIGURE S1 The toxicity rest of canthin-6-one.

TABLE S1 The basic information of ten positive compounds from screening result.

DATA SHEET S1 All western blot raw data.
