# Supplementary figures and images for: Canthin-6-One Accelerates Alpha-Synuclein Degradation by Enhancing UPS Activity: Drug Target Identification by CRISPR-Cas9 Whole Genome-Wide Screening Technology
Source: Front Pharmacol. 2019 Jan 28;10:16. doi: 10.3389/fphar.2019.00016 (PMC6360163; doi:10.3389/fphar.2019.00016)

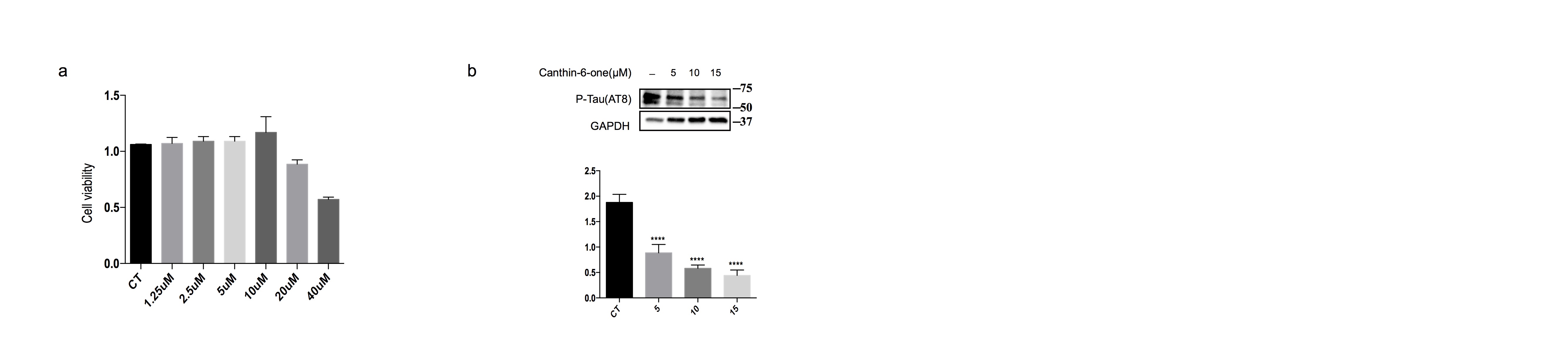

Supplement: FIGURE S1 — The toxicity rest of canthin-6-one. [file Image_1.JPEG]

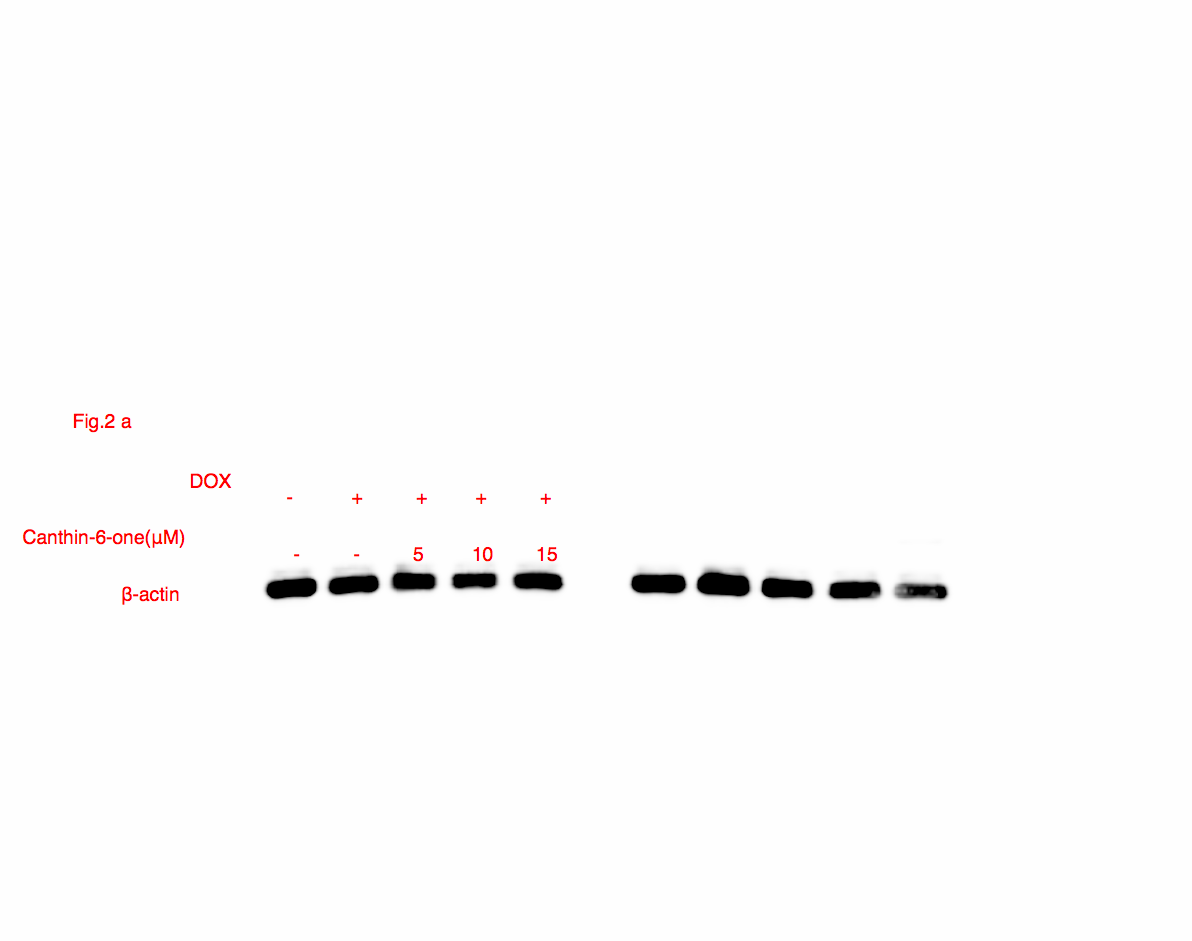

Supplement: DATA SHEET S1 — All western blot raw data. [file Data_Sheet_1.ZIP › wb origin/fig.2/1.A53T DOS/┬╢┬í-syn(A53T) actin dos.tif.tif]

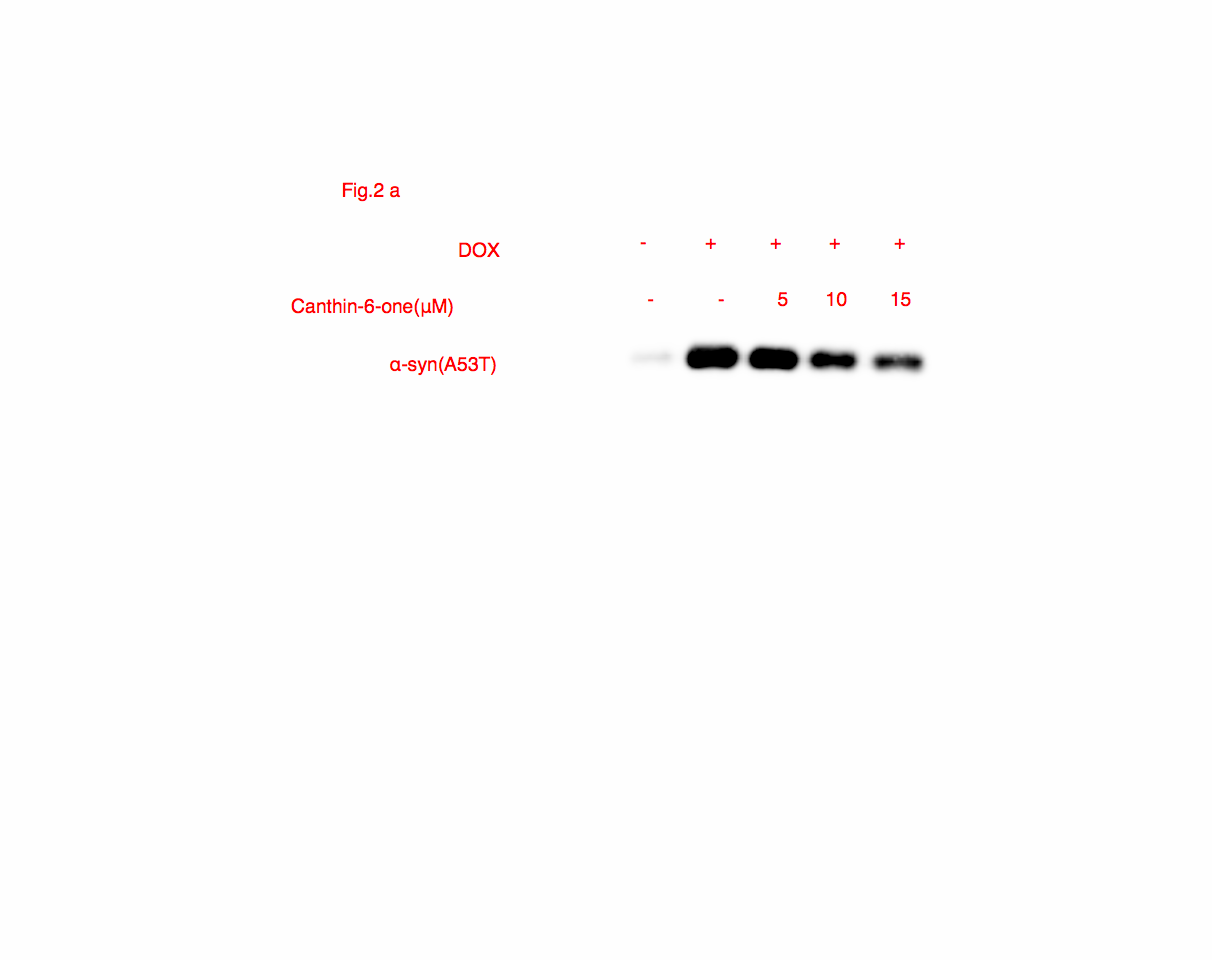

Supplement: DATA SHEET S1 — All western blot raw data. [file Data_Sheet_1.ZIP › wb origin/fig.2/1.A53T DOS/┬╢┬í-syn(A53T) dos.tif]

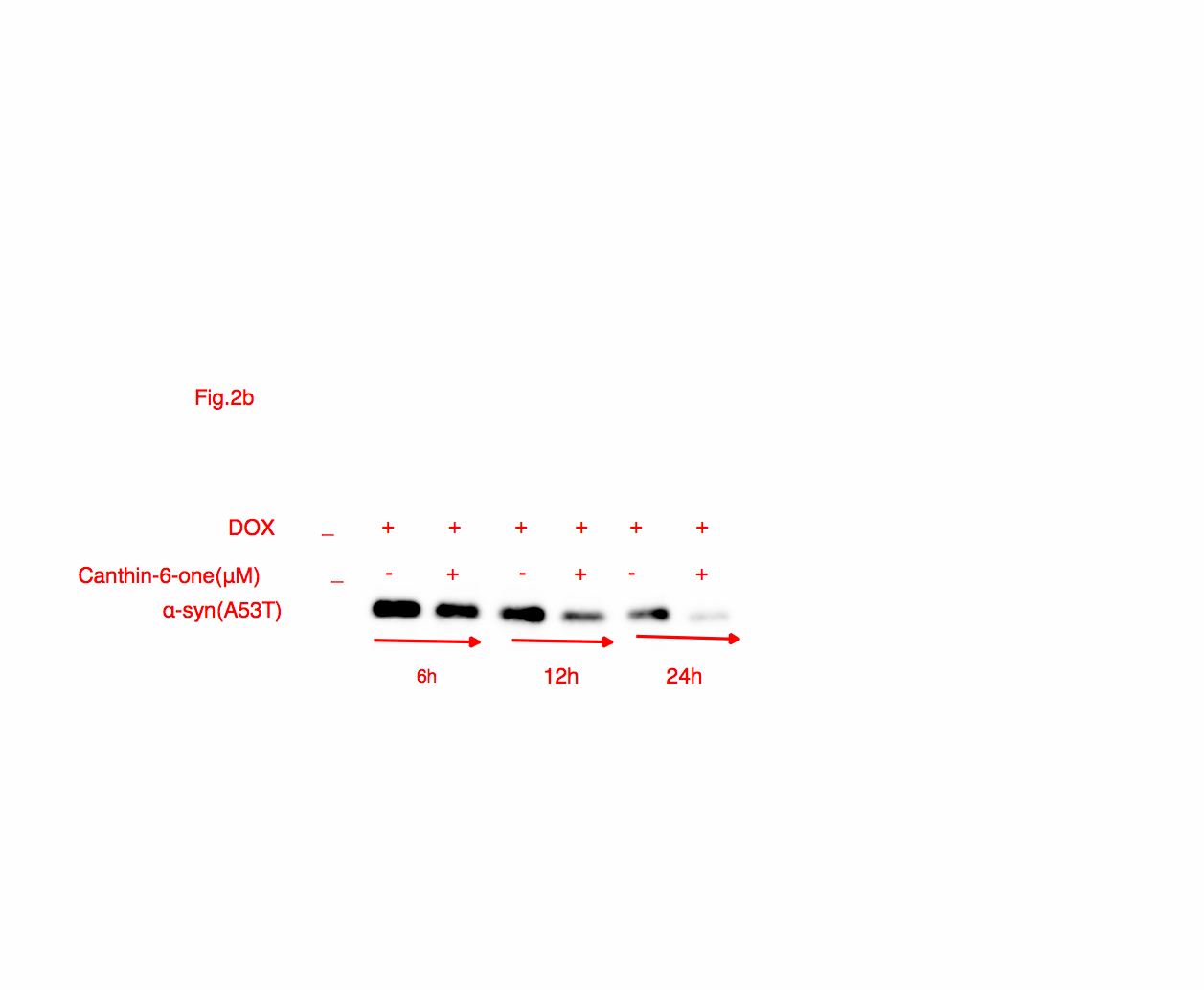

Supplement: DATA SHEET S1 — All western blot raw data. [file Data_Sheet_1.ZIP › wb origin/fig.2/2.A53t time/asyn a53t.tif]

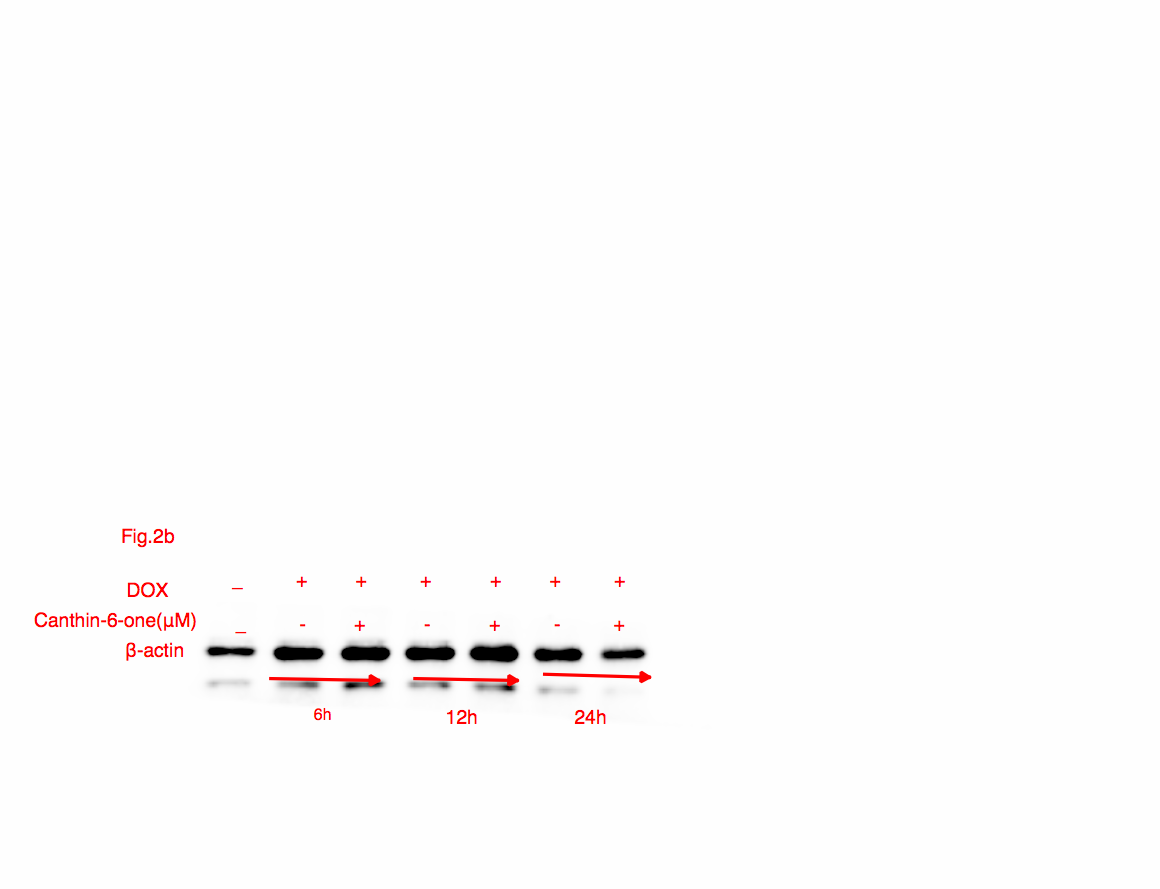

Supplement: DATA SHEET S1 — All western blot raw data. [file Data_Sheet_1.ZIP › wb origin/fig.2/2.A53t time/┬╢┬1⁄4-actin.tif]

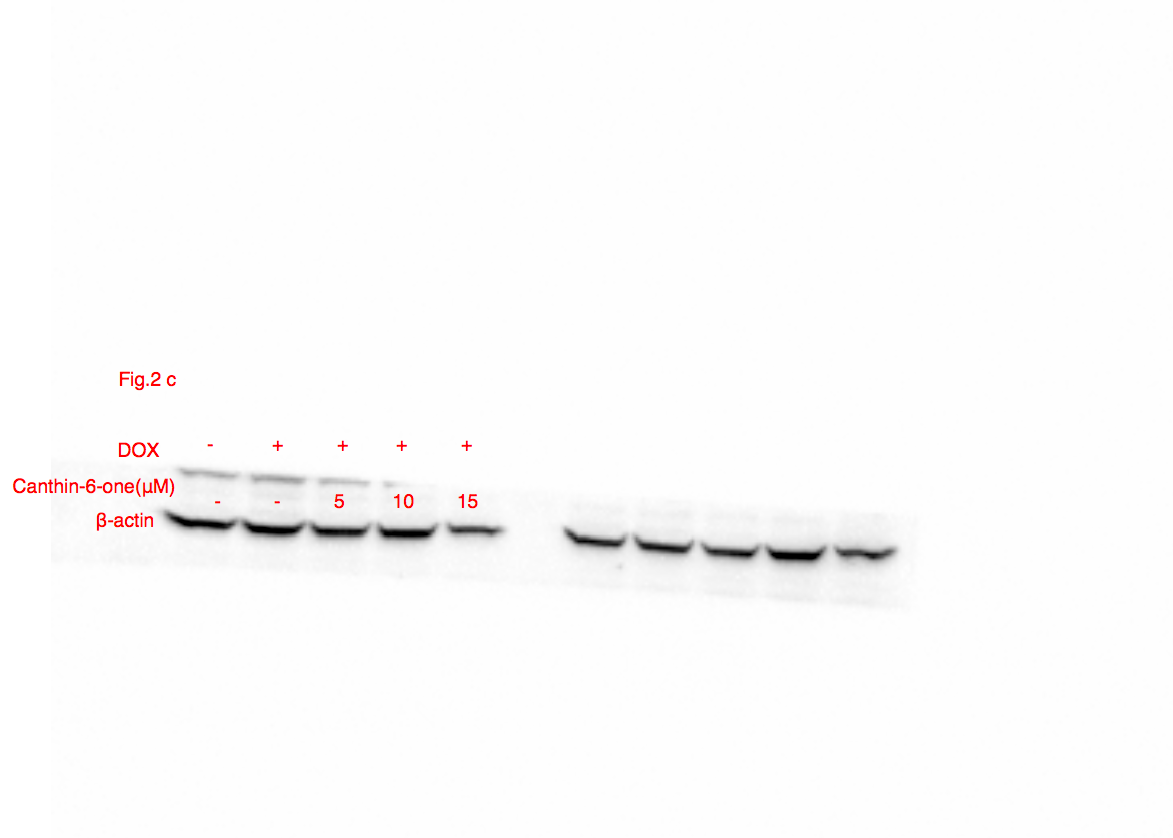

Supplement: DATA SHEET S1 — All western blot raw data. [file Data_Sheet_1.ZIP › wb origin/fig.2/3.WT dos/ACTINscn.tif]

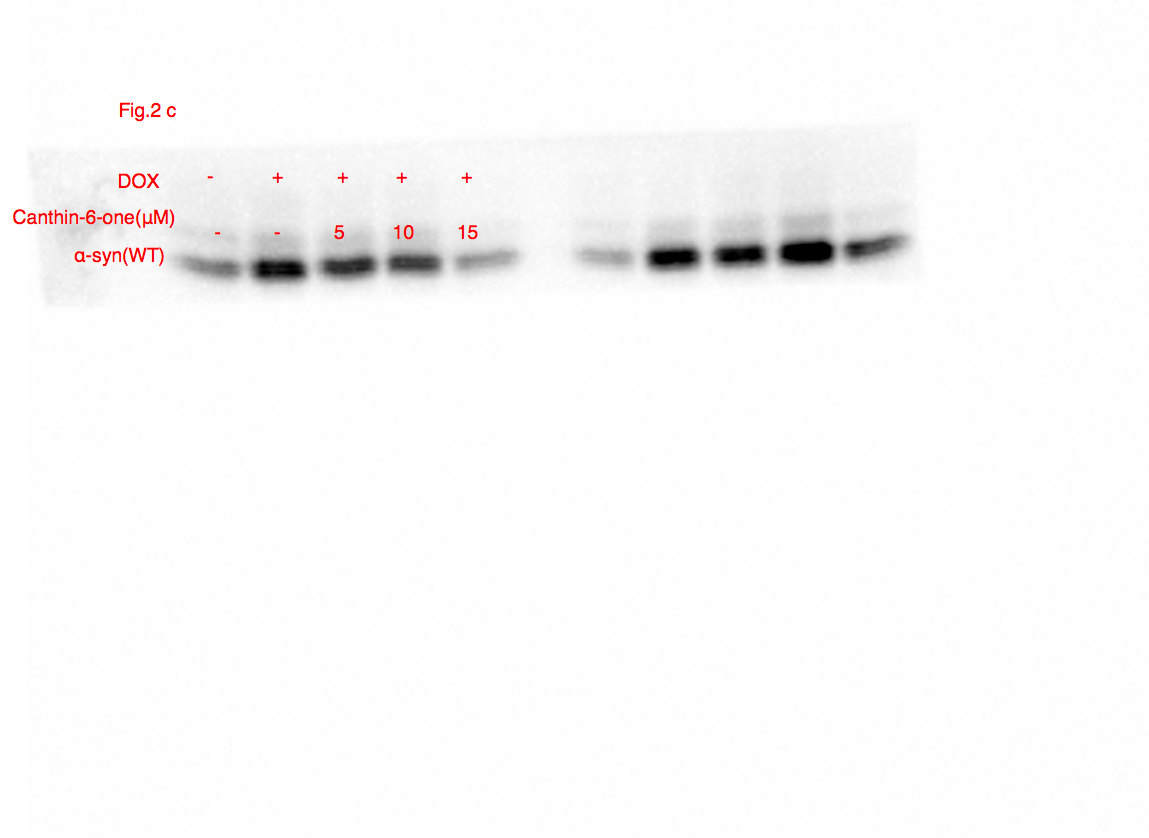

Supplement: DATA SHEET S1 — All western blot raw data. [file Data_Sheet_1.ZIP › wb origin/fig.2/3.WT dos/┬╢┬í-syn(WT).tif]

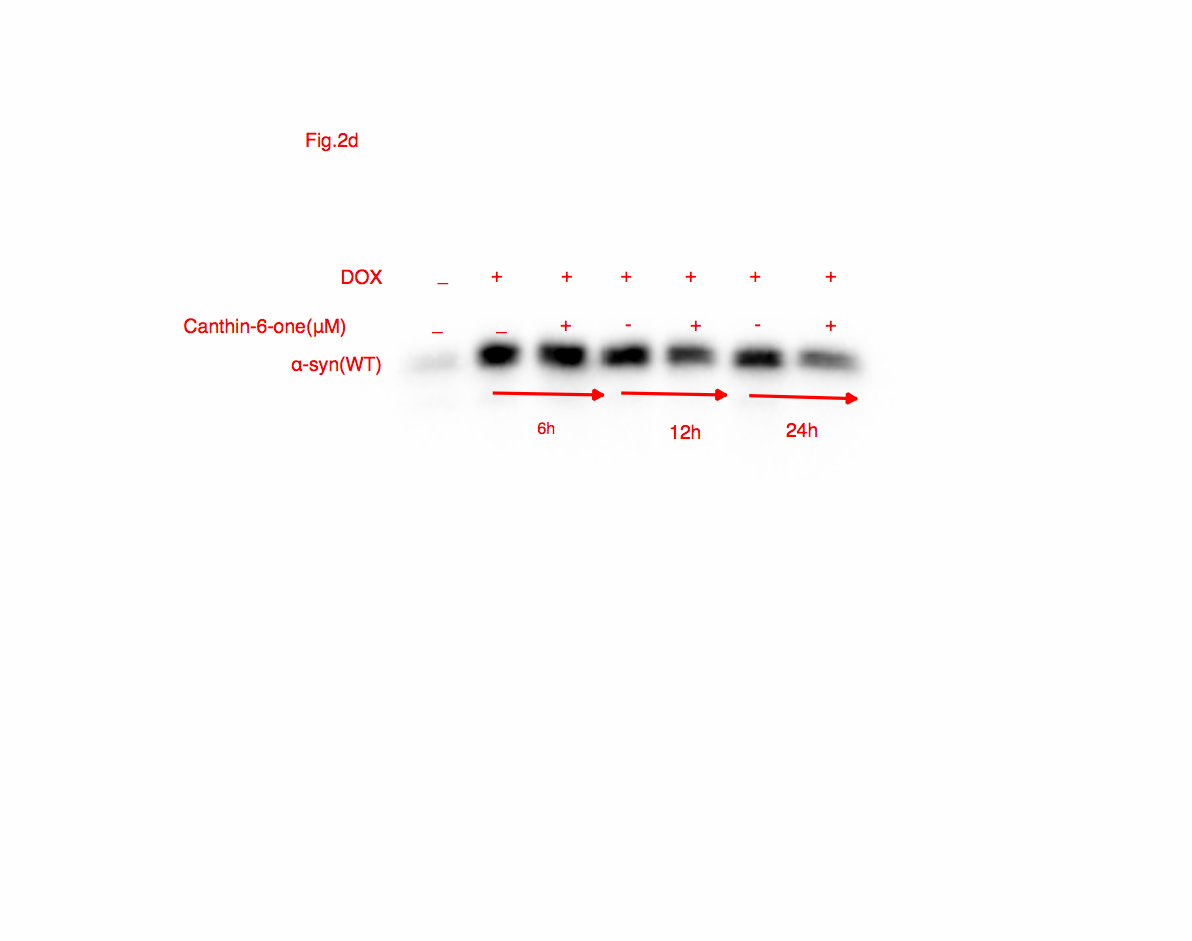

Supplement: DATA SHEET S1 — All western blot raw data. [file Data_Sheet_1.ZIP › wb origin/fig.2/4.WT TIME/asyn wt time.tif]

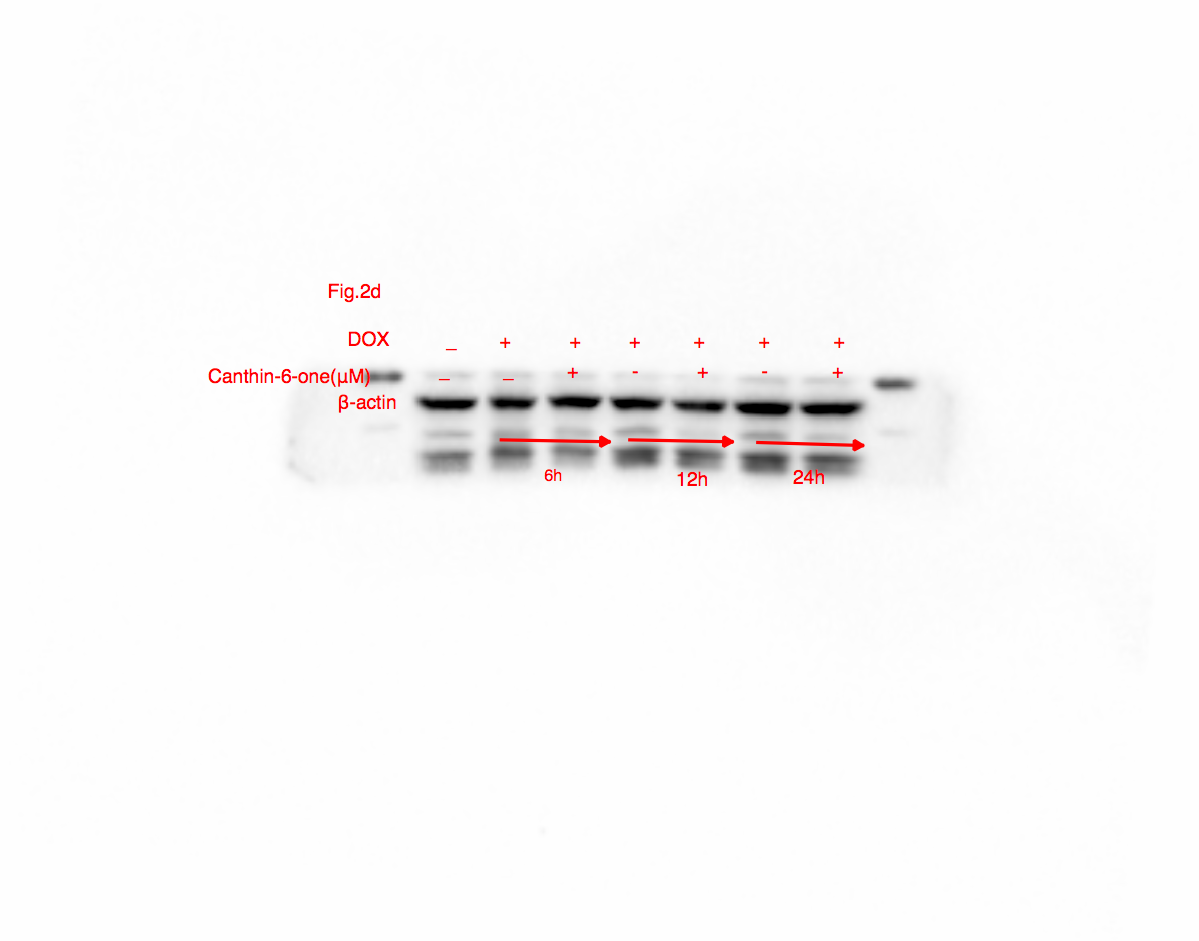

Supplement: DATA SHEET S1 — All western blot raw data. [file Data_Sheet_1.ZIP › wb origin/fig.2/4.WT TIME/┬╢┬1⁄4-actin.tif]

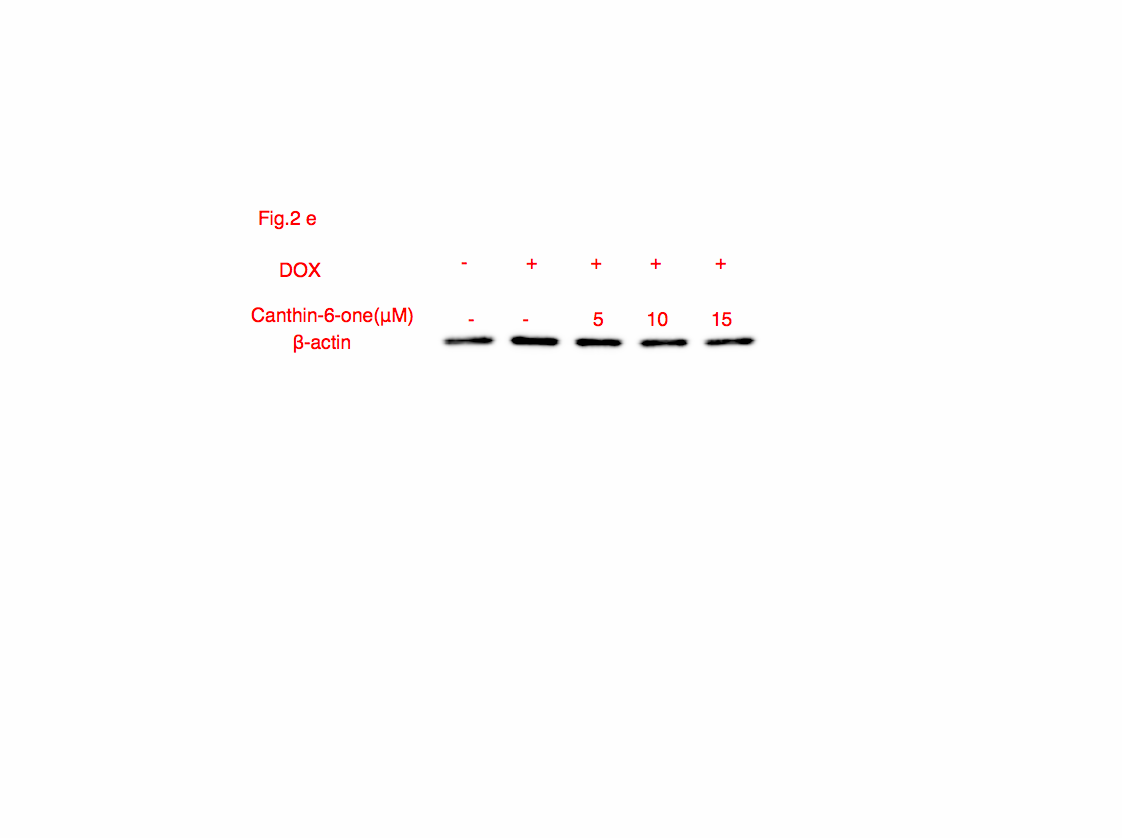

Supplement: DATA SHEET S1 — All western blot raw data. [file Data_Sheet_1.ZIP › wb origin/fig.2/5.A30P DOS/actin.tif]

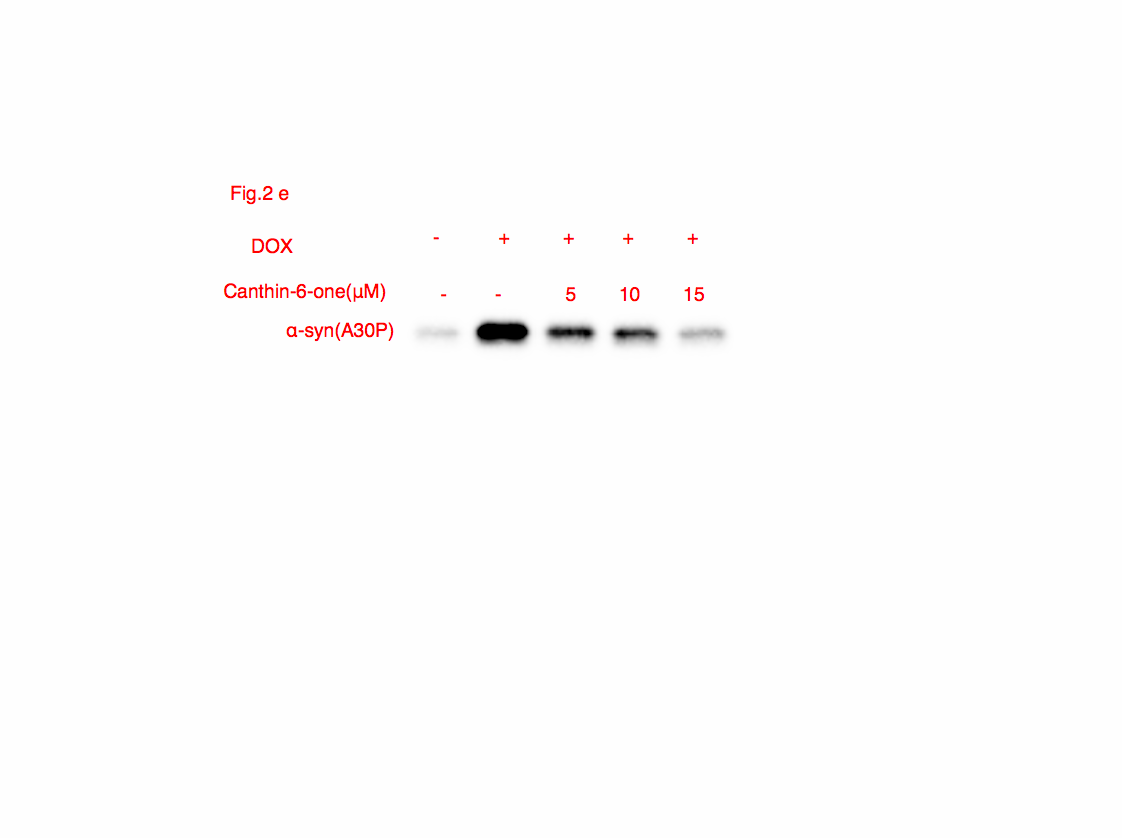

Supplement: DATA SHEET S1 — All western blot raw data. [file Data_Sheet_1.ZIP › wb origin/fig.2/5.A30P DOS/┬╢┬í-syn(A30P).tif]

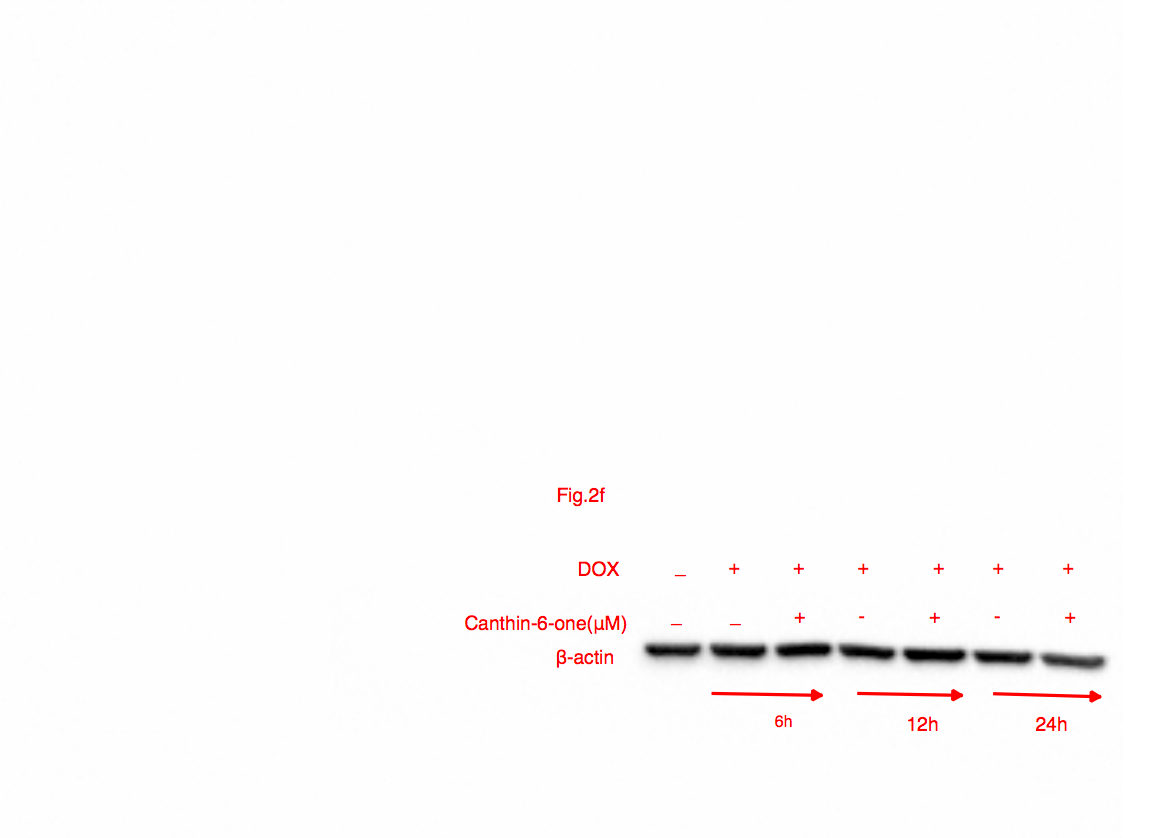

Supplement: DATA SHEET S1 — All western blot raw data. [file Data_Sheet_1.ZIP › wb origin/fig.2/6.a30p time/a30p actin.tif]

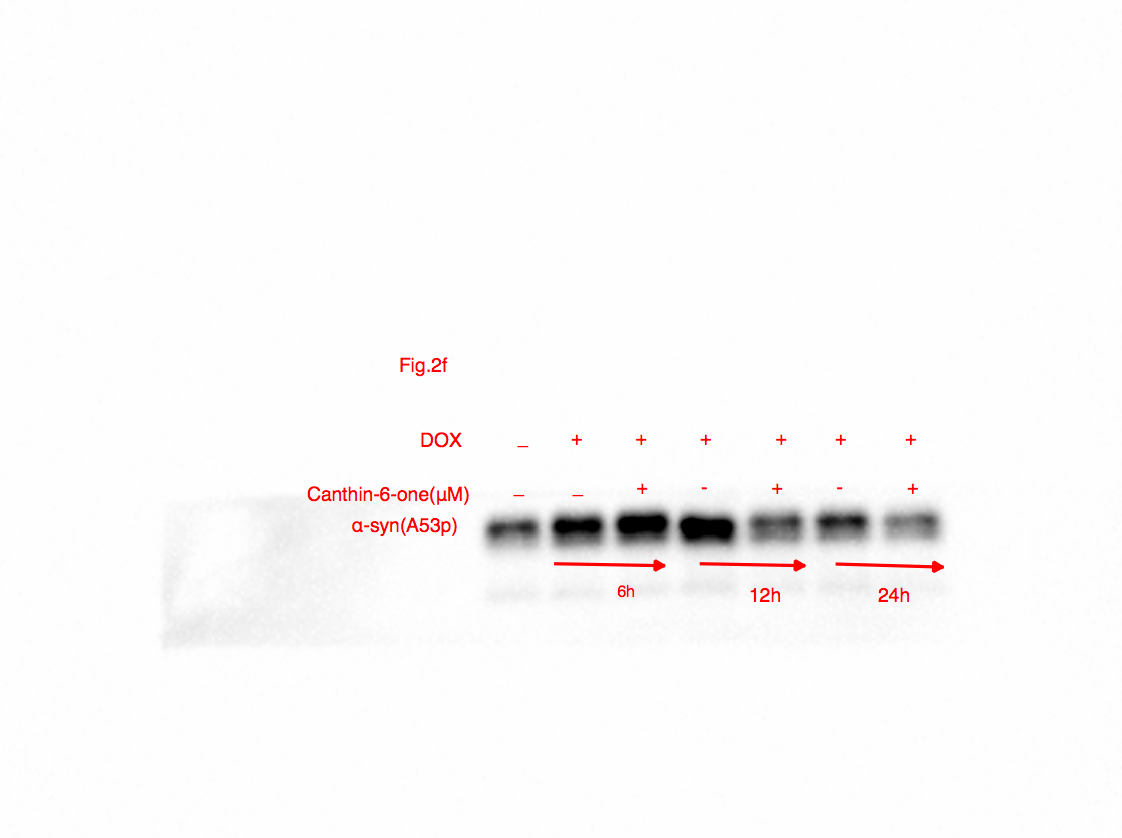

Supplement: DATA SHEET S1 — All western blot raw data. [file Data_Sheet_1.ZIP › wb origin/fig.2/6.a30p time/a30p.tif]

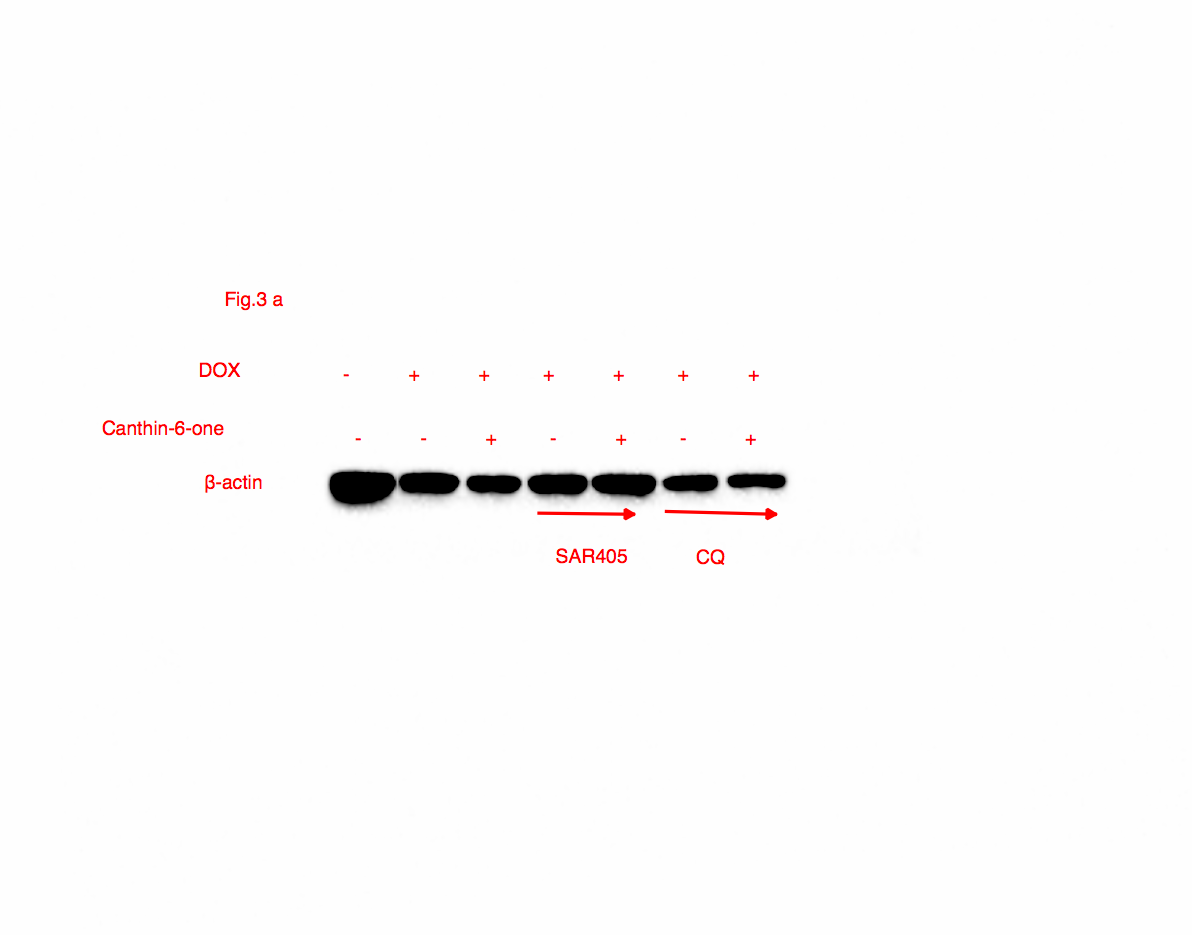

Supplement: DATA SHEET S1 — All western blot raw data. [file Data_Sheet_1.ZIP › wb origin/fig.3/ autophagy inhibitor/actin.tif]

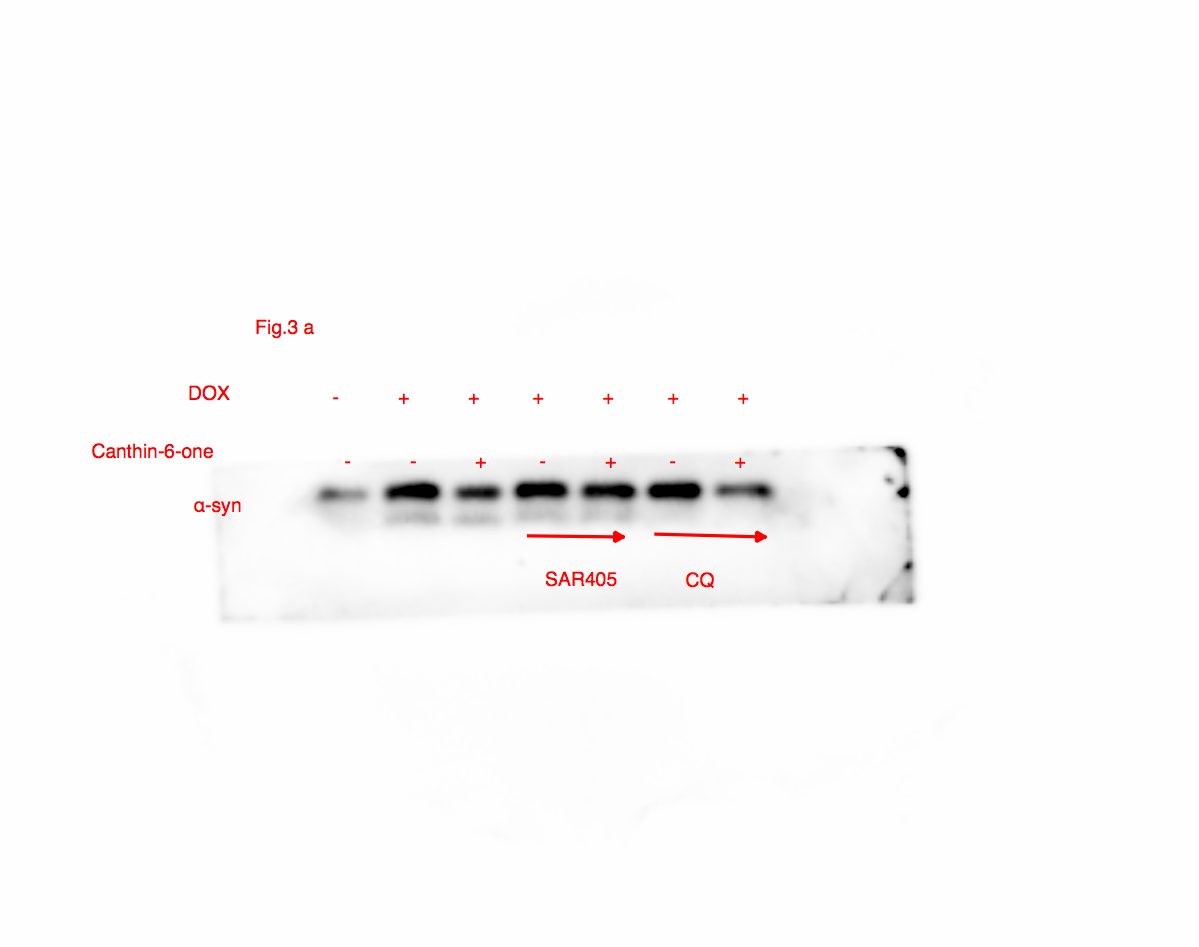

Supplement: DATA SHEET S1 — All western blot raw data. [file Data_Sheet_1.ZIP › wb origin/fig.3/ autophagy inhibitor/asyn..tif]

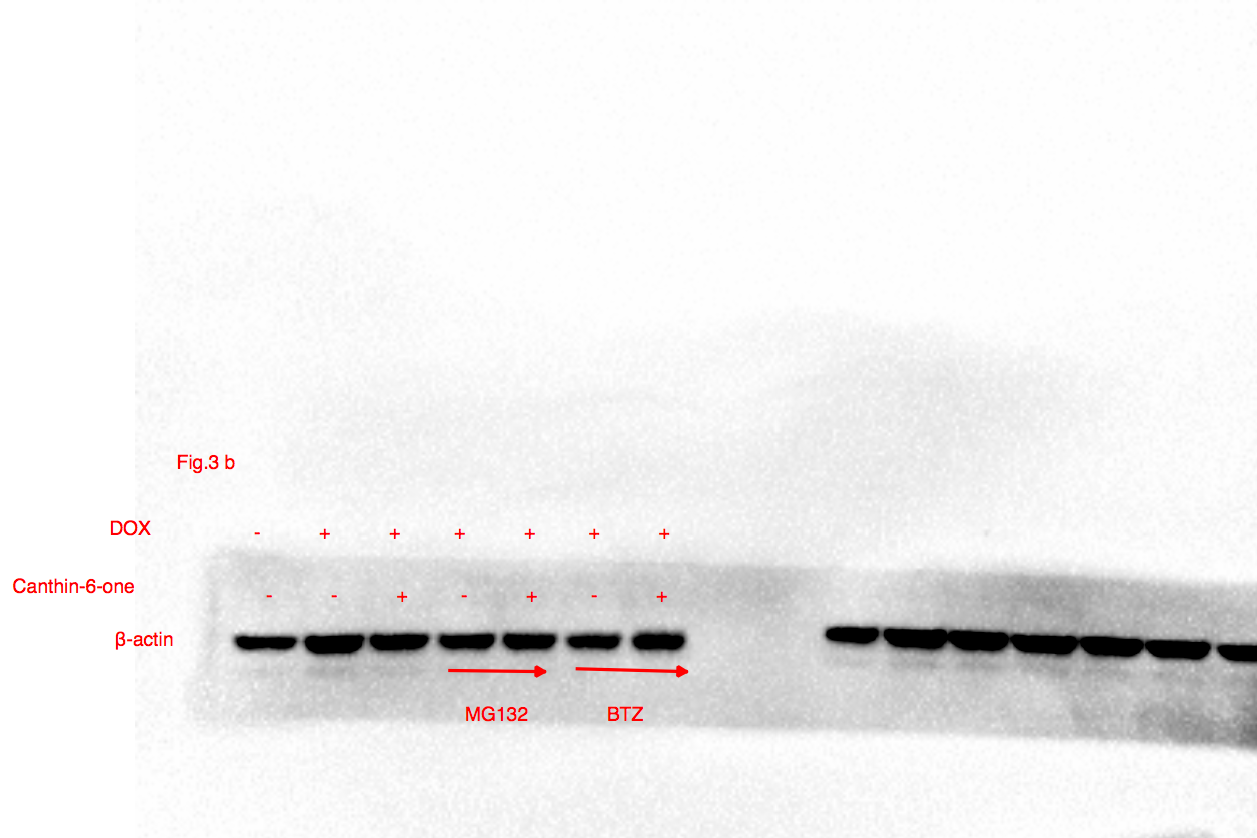

Supplement: DATA SHEET S1 — All western blot raw data. [file Data_Sheet_1.ZIP › wb origin/fig.3/ ups inhibitor/actinscn.tif]

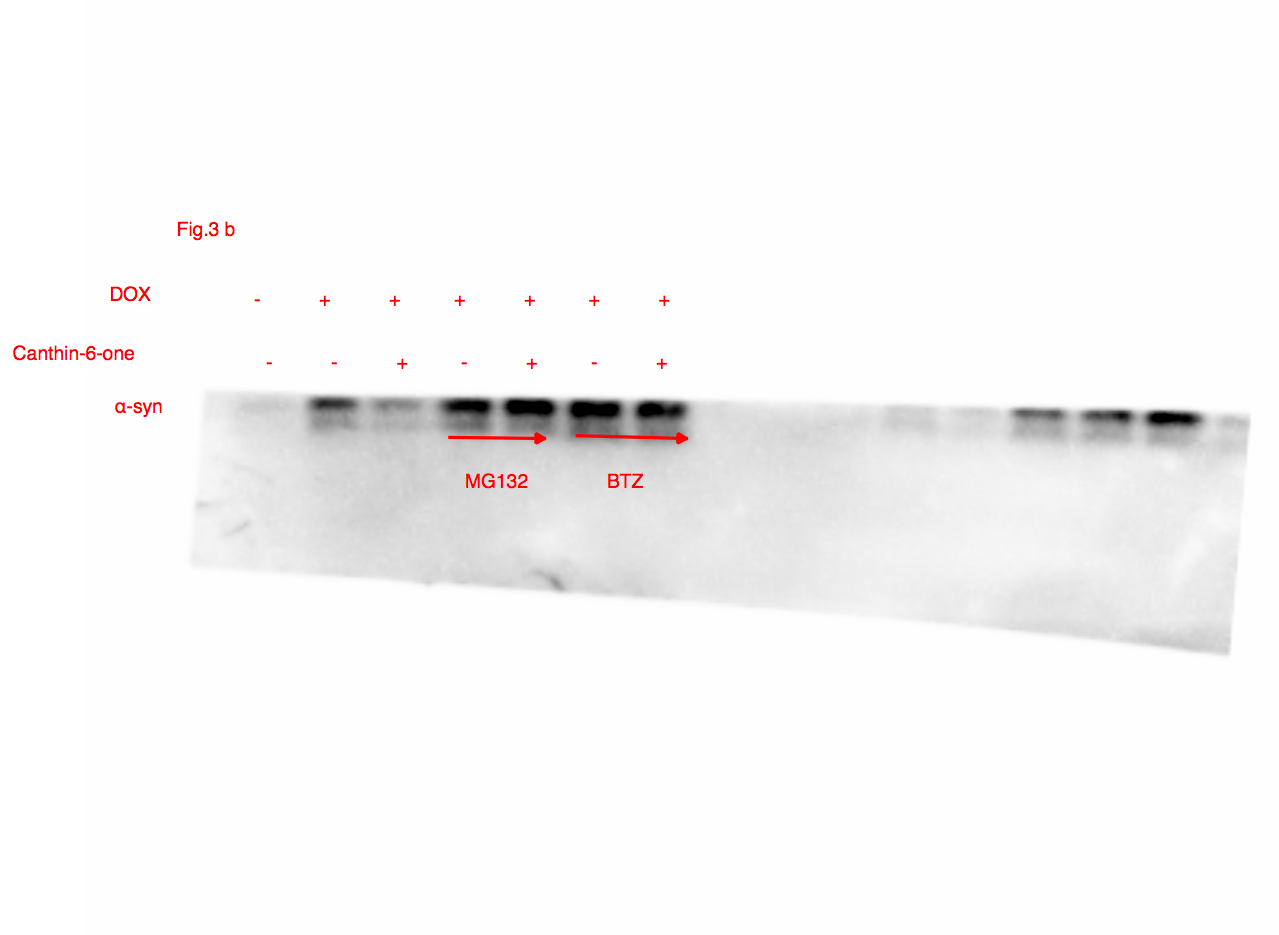

Supplement: DATA SHEET S1 — All western blot raw data. [file Data_Sheet_1.ZIP › wb origin/fig.3/ ups inhibitor/asyn..tif]

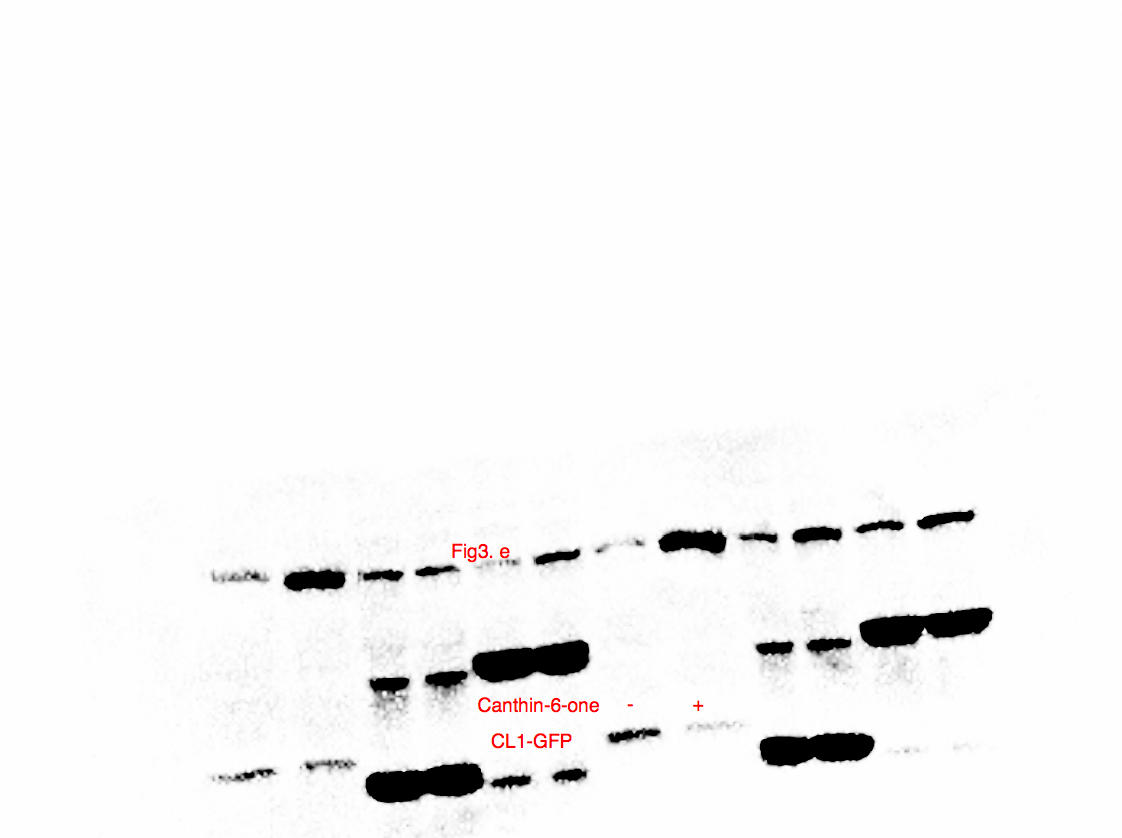

Supplement: DATA SHEET S1 — All western blot raw data. [file Data_Sheet_1.ZIP › wb origin/fig.3/CL-1/cl1 .tif]

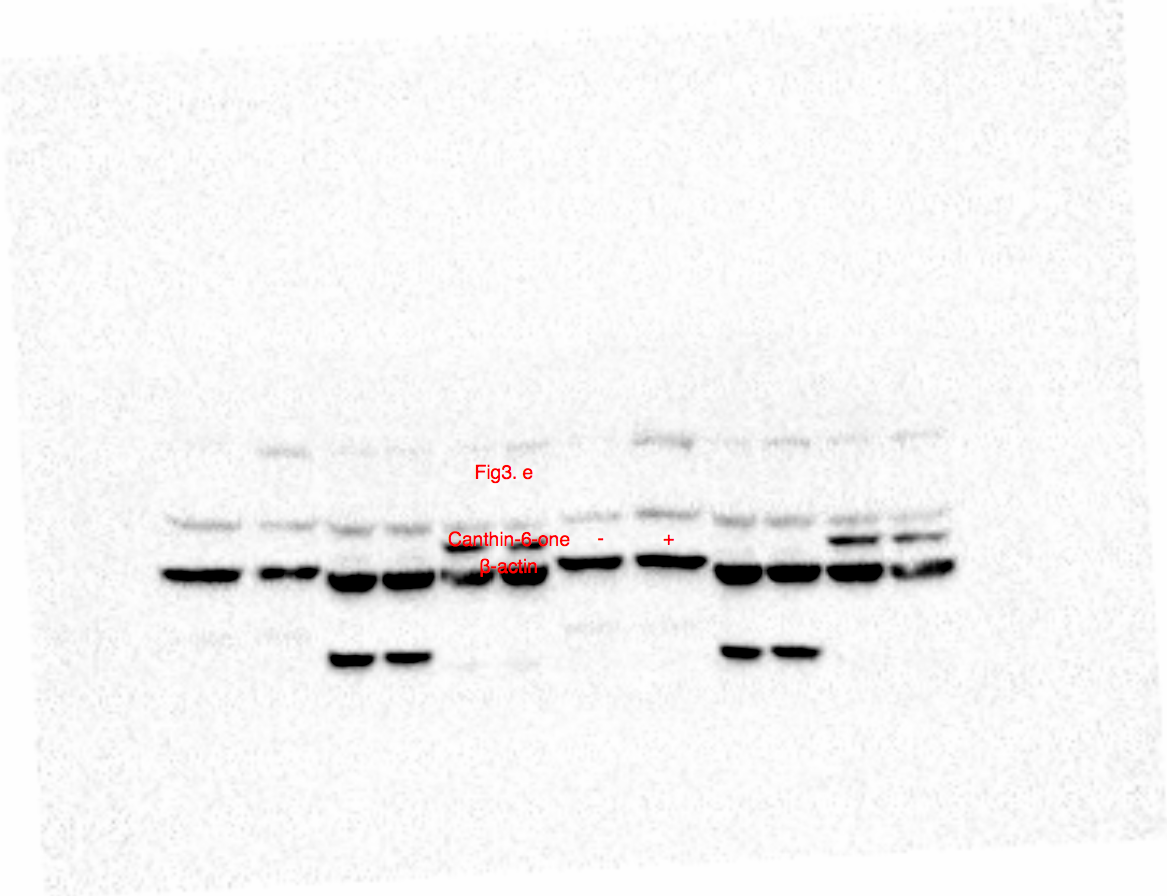

Supplement: DATA SHEET S1 — All western blot raw data. [file Data_Sheet_1.ZIP › wb origin/fig.3/CL-1/cl1 actin.tif]

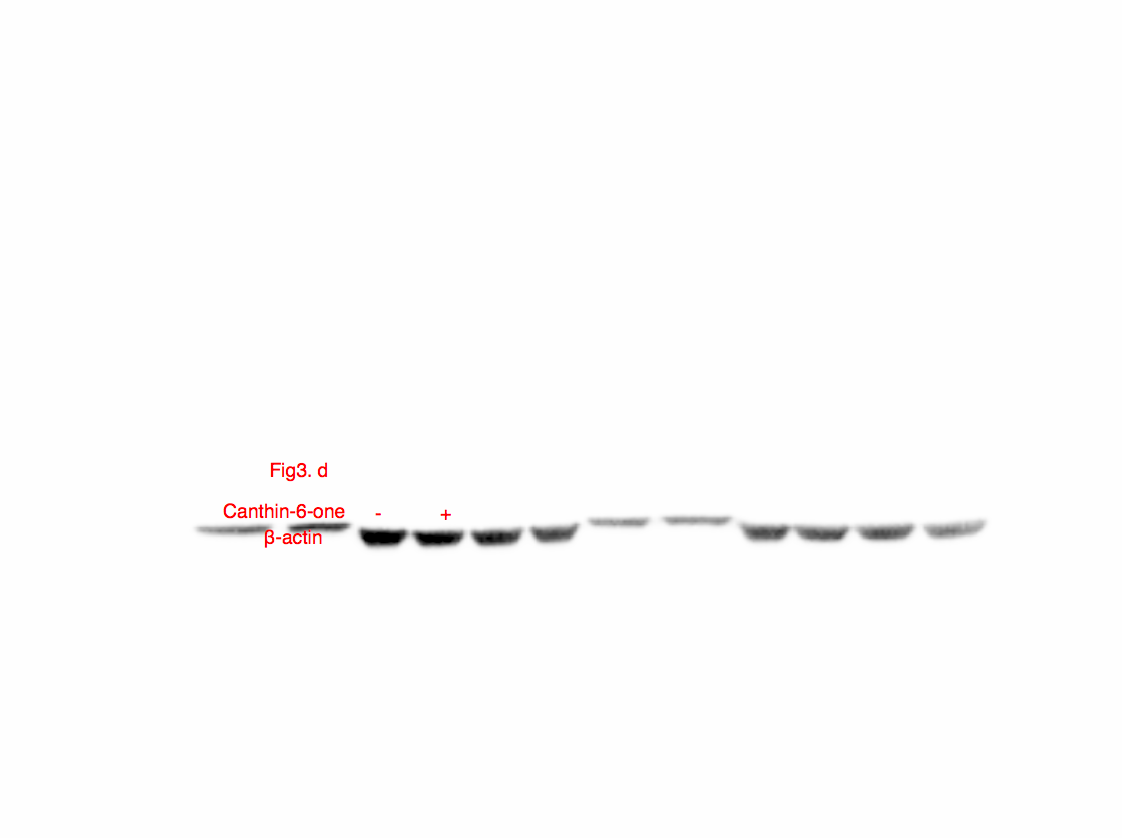

Supplement: DATA SHEET S1 — All western blot raw data. [file Data_Sheet_1.ZIP › wb origin/fig.3/Ub-G76V-GFP/Ub-G76V-GFP actin.tif]

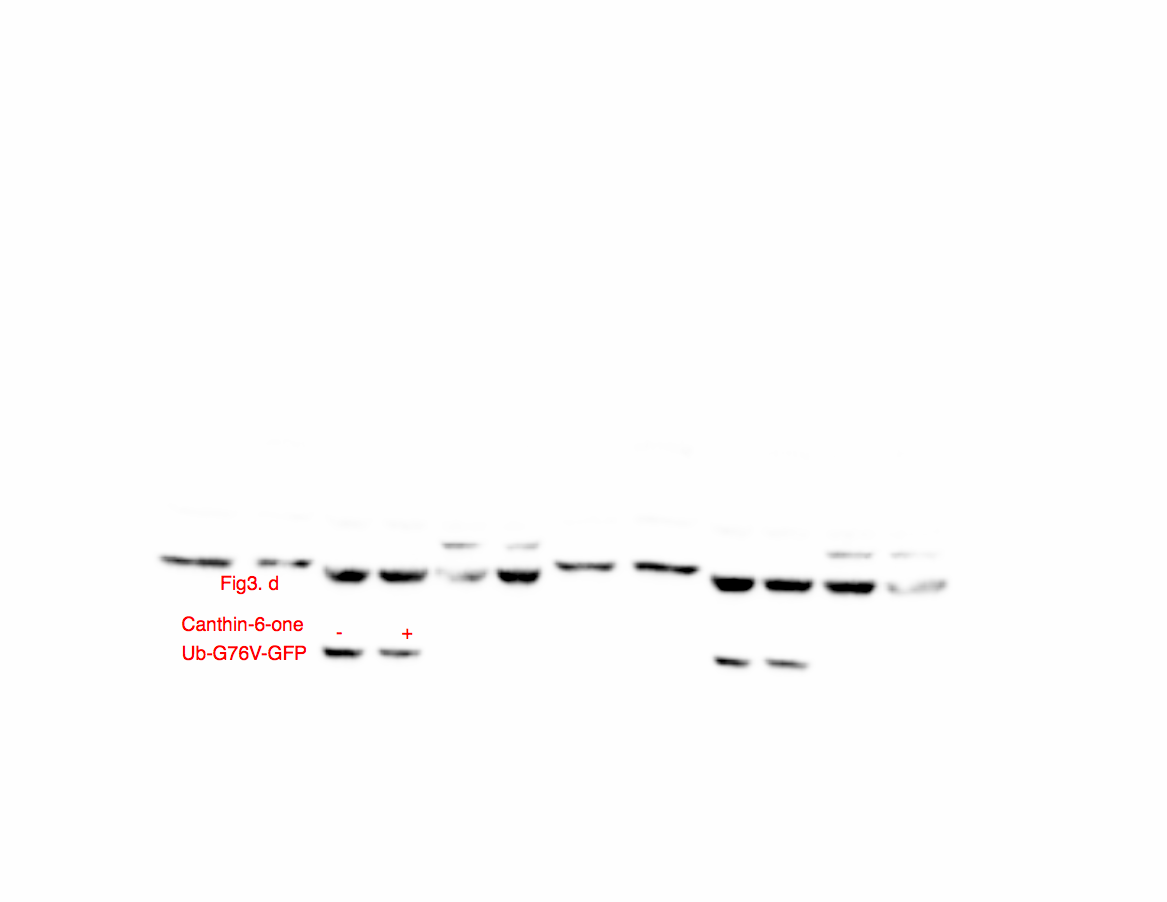

Supplement: DATA SHEET S1 — All western blot raw data. [file Data_Sheet_1.ZIP › wb origin/fig.3/Ub-G76V-GFP/Ub-G76V-GFP.tif]

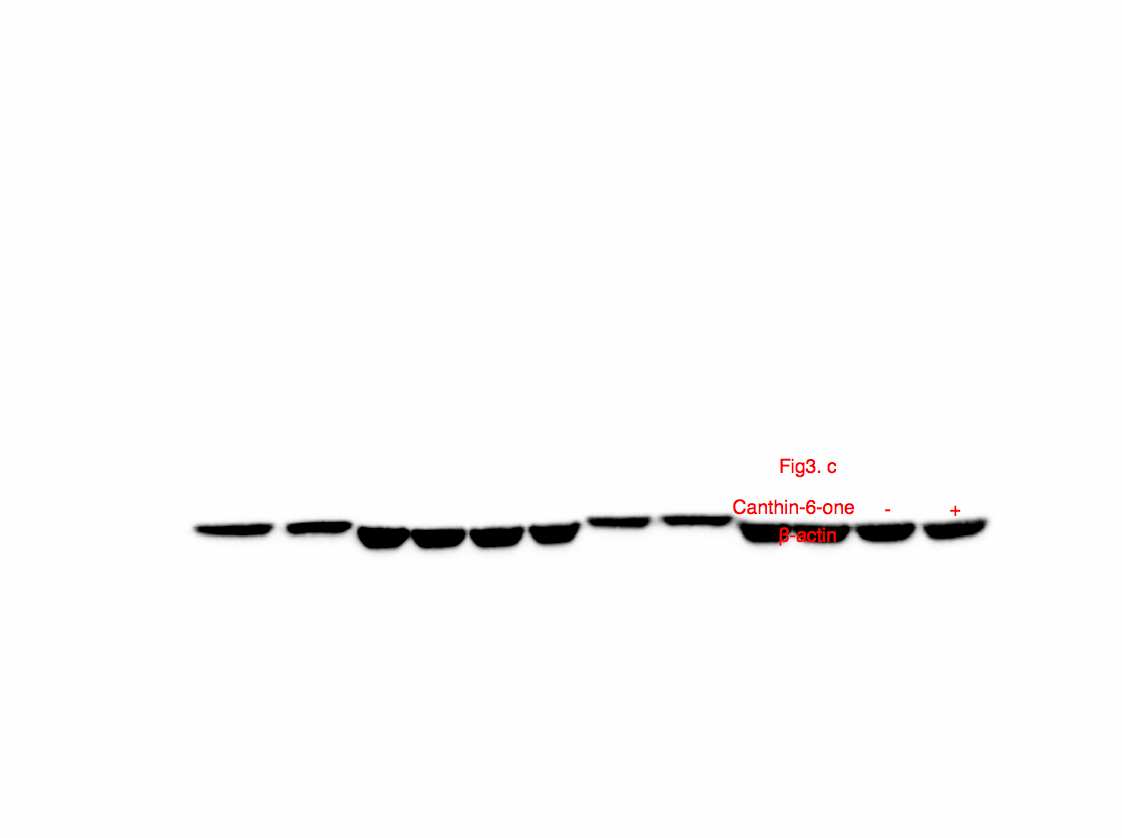

Supplement: DATA SHEET S1 — All western blot raw data. [file Data_Sheet_1.ZIP › wb origin/fig.3/Ub-R-GFP/Ub-R-GFP actin.tif]

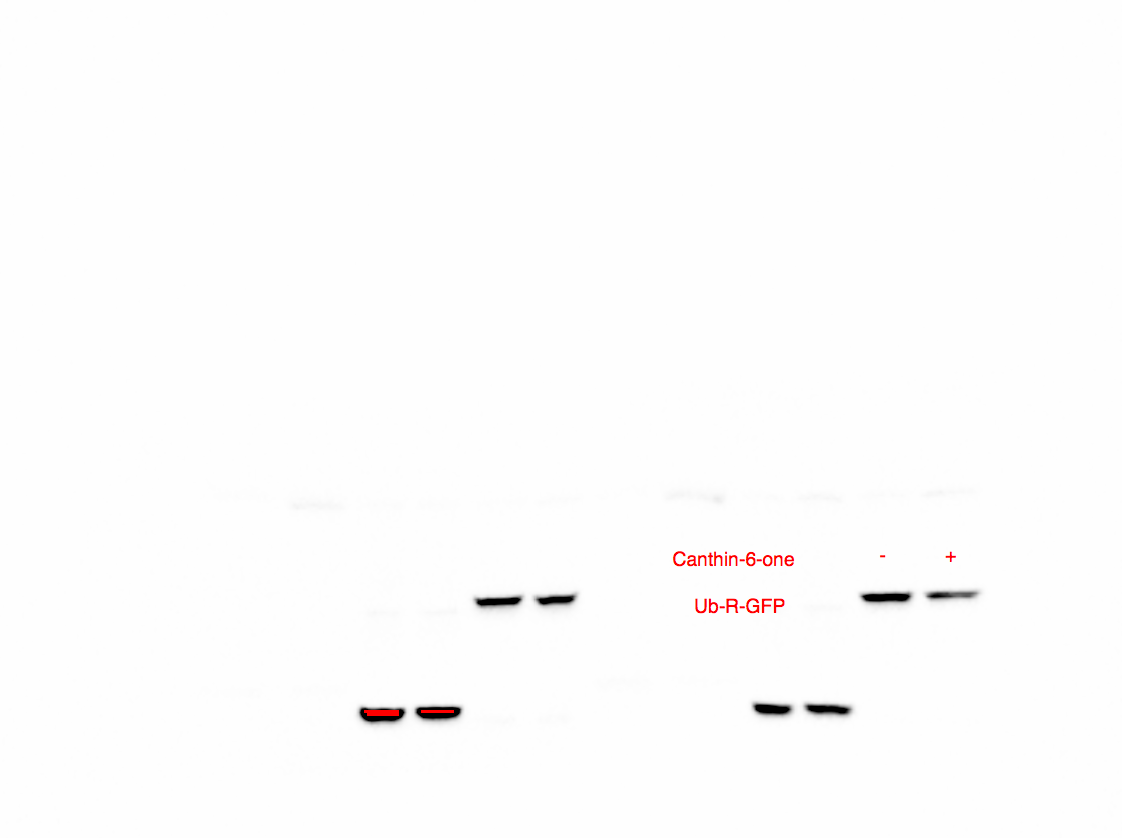

Supplement: DATA SHEET S1 — All western blot raw data. [file Data_Sheet_1.ZIP › wb origin/fig.3/Ub-R-GFP/Ub-R-GFP.tif]

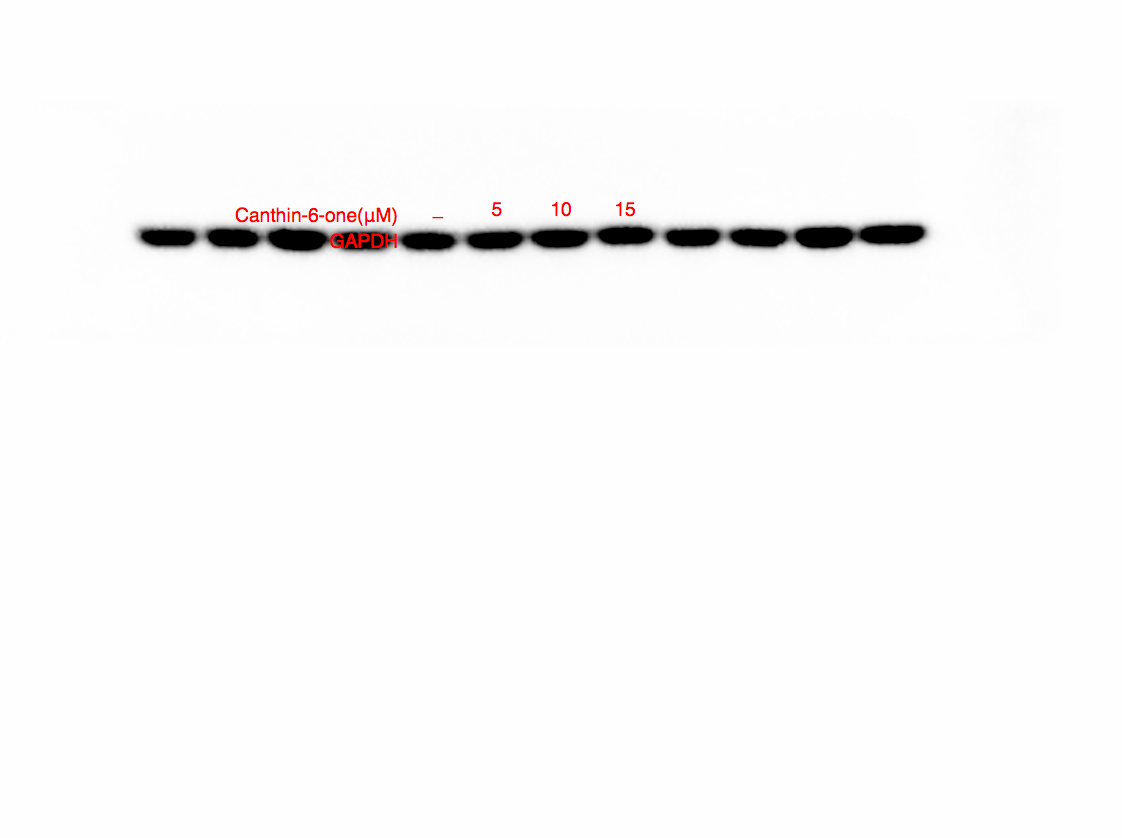

Supplement: DATA SHEET S1 — All western blot raw data. [file Data_Sheet_1.ZIP › wb origin/fig.5/b/GAPDH scn.tif]

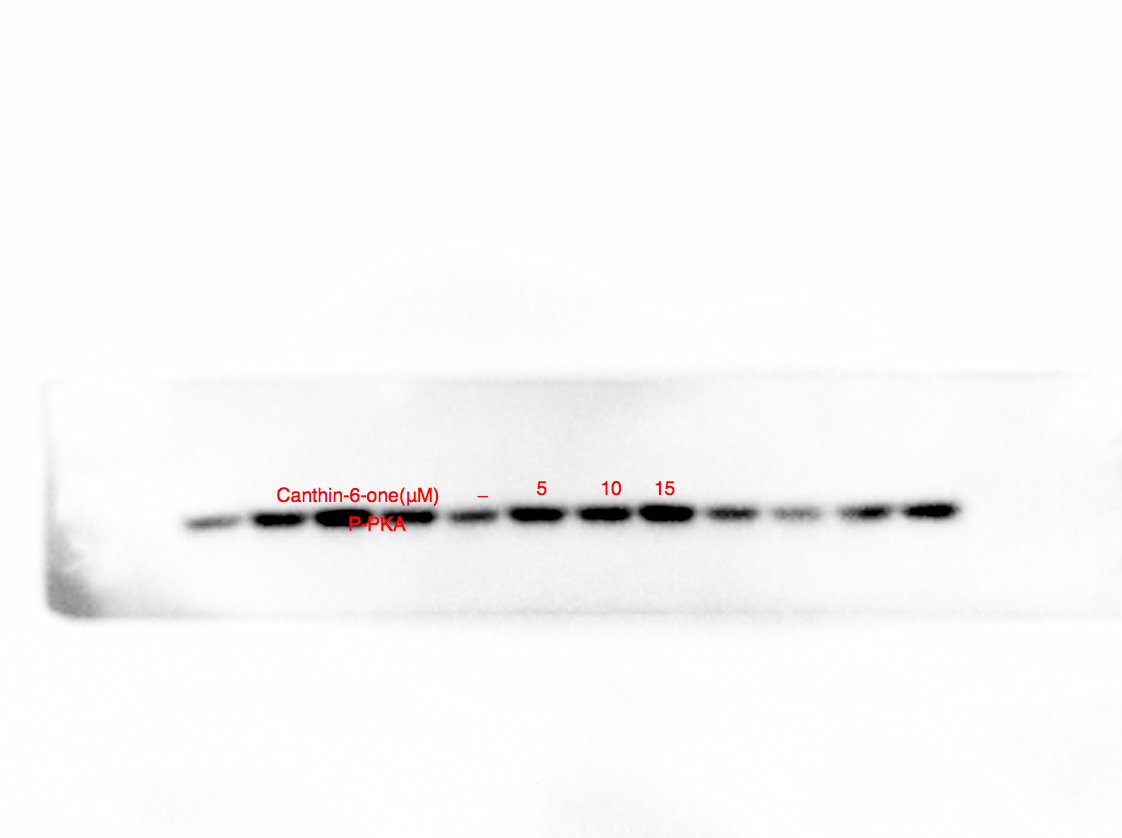

Supplement: DATA SHEET S1 — All western blot raw data. [file Data_Sheet_1.ZIP › wb origin/fig.5/b/P-PKA.tif]

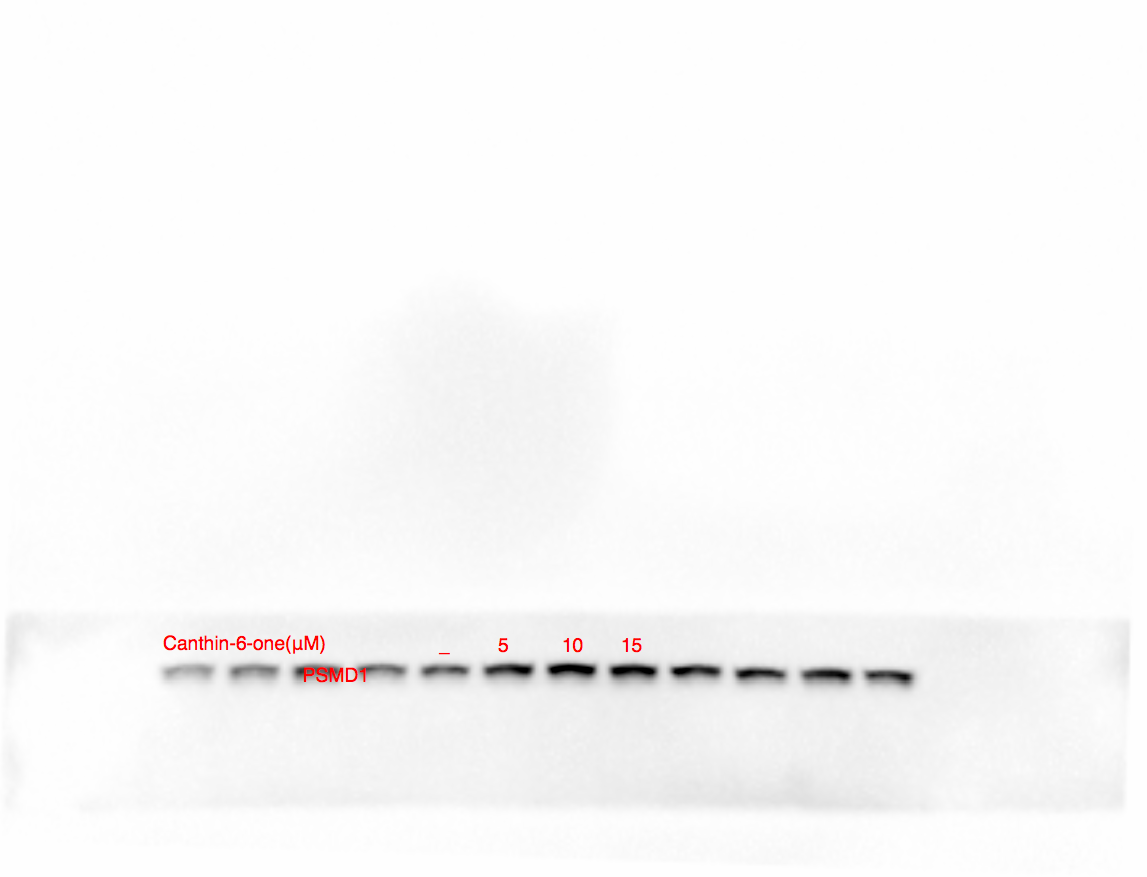

Supplement: DATA SHEET S1 — All western blot raw data. [file Data_Sheet_1.ZIP › wb origin/fig.5/b/PSMD1.tif]

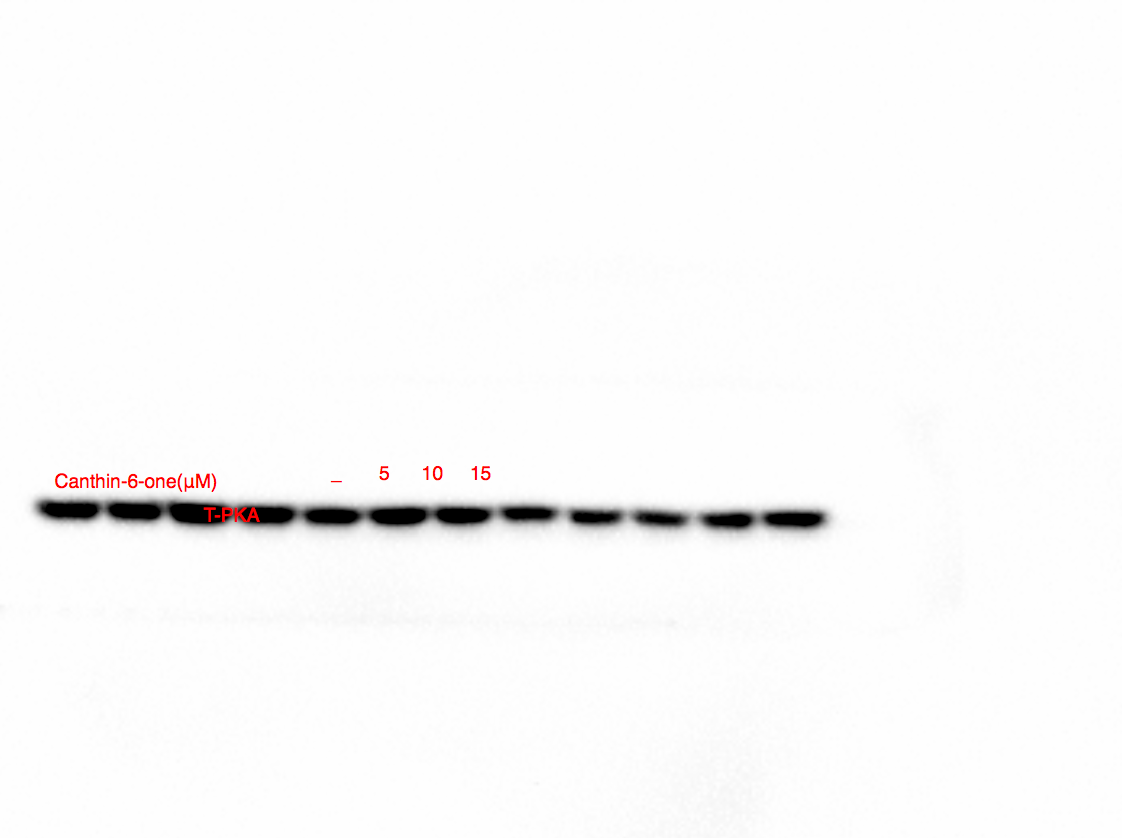

Supplement: DATA SHEET S1 — All western blot raw data. [file Data_Sheet_1.ZIP › wb origin/fig.5/b/T-PKA.tif]

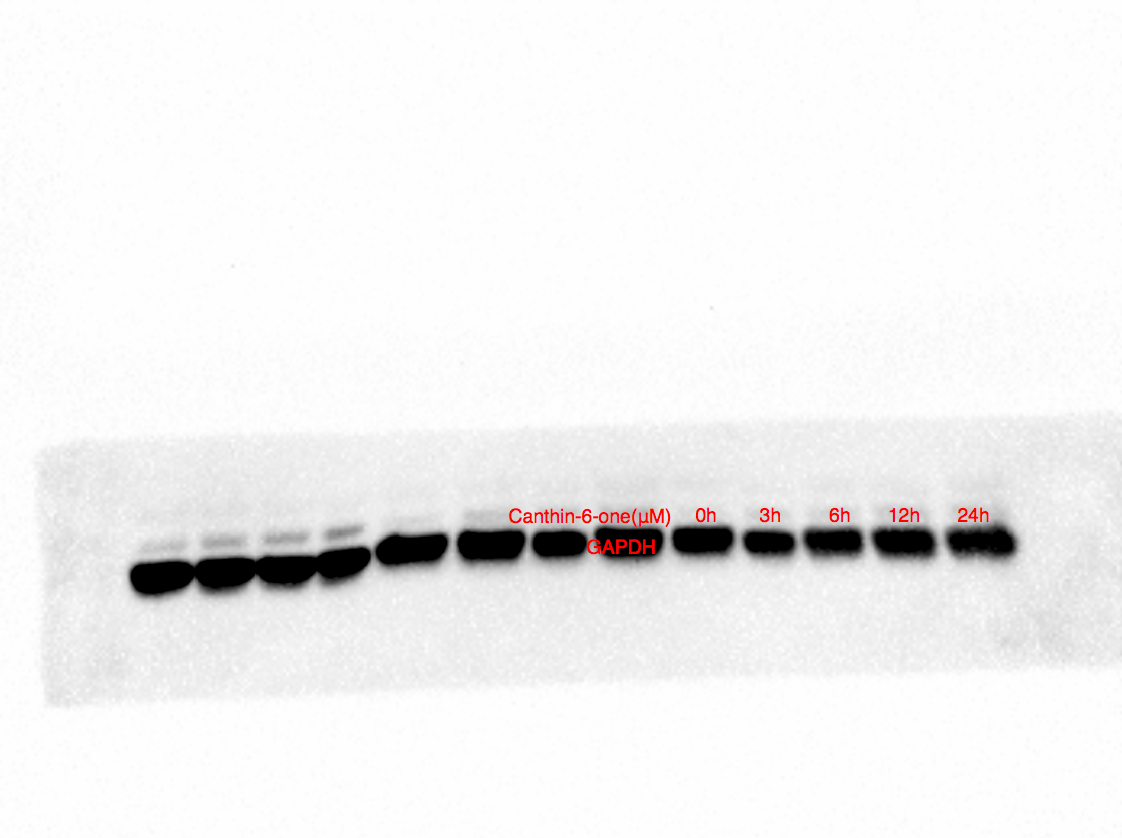

Supplement: DATA SHEET S1 — All western blot raw data. [file Data_Sheet_1.ZIP › wb origin/fig.5/c/GAPDH.tif]

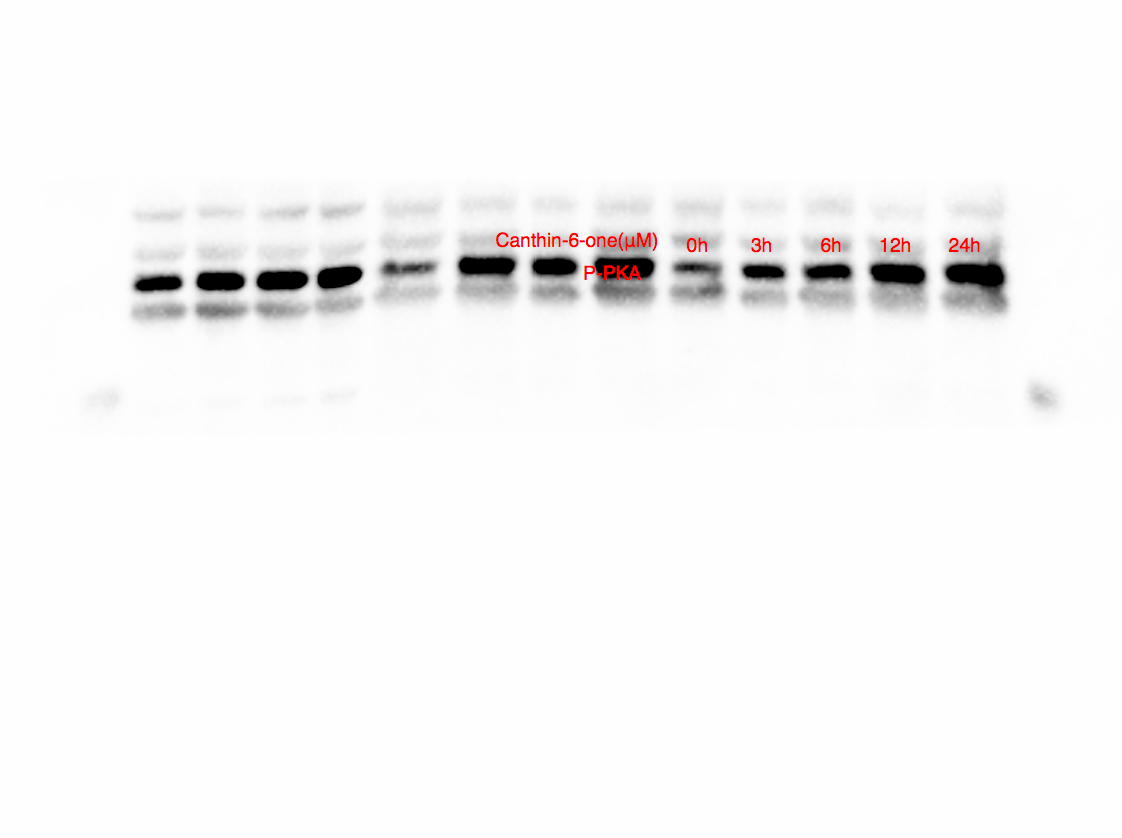

Supplement: DATA SHEET S1 — All western blot raw data. [file Data_Sheet_1.ZIP › wb origin/fig.5/c/P-PKA.tif]

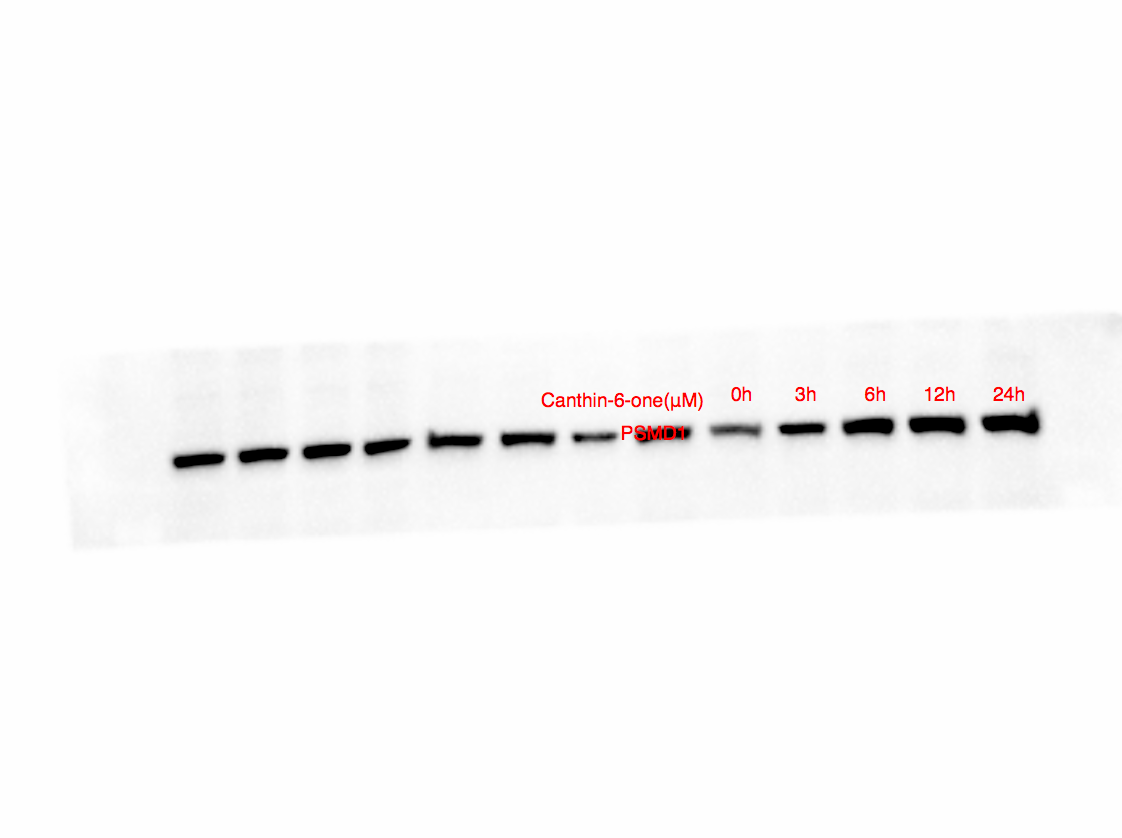

Supplement: DATA SHEET S1 — All western blot raw data. [file Data_Sheet_1.ZIP › wb origin/fig.5/c/PSMD1.tif]

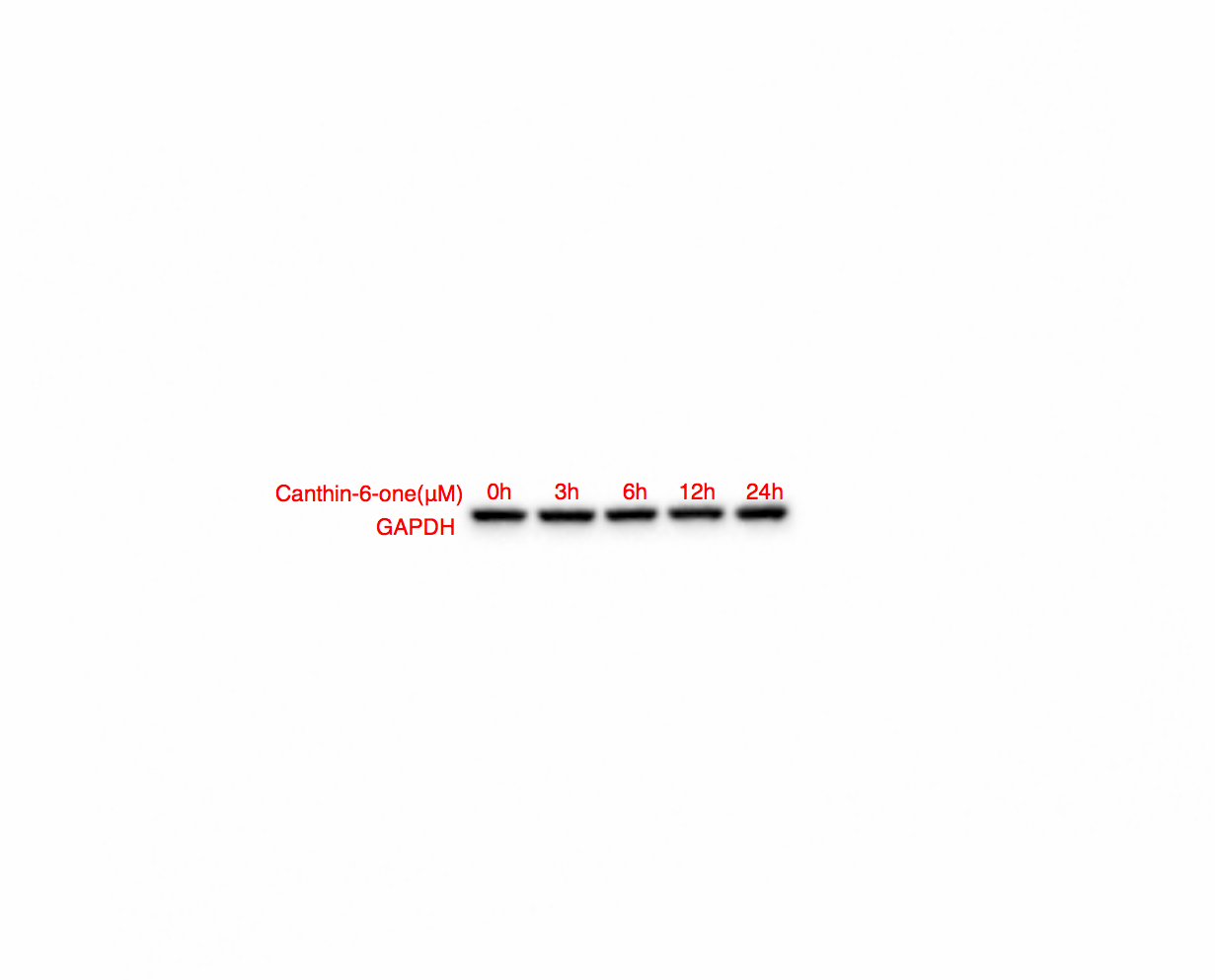

Supplement: DATA SHEET S1 — All western blot raw data. [file Data_Sheet_1.ZIP › wb origin/fig.5/c/T-PKA.tif]

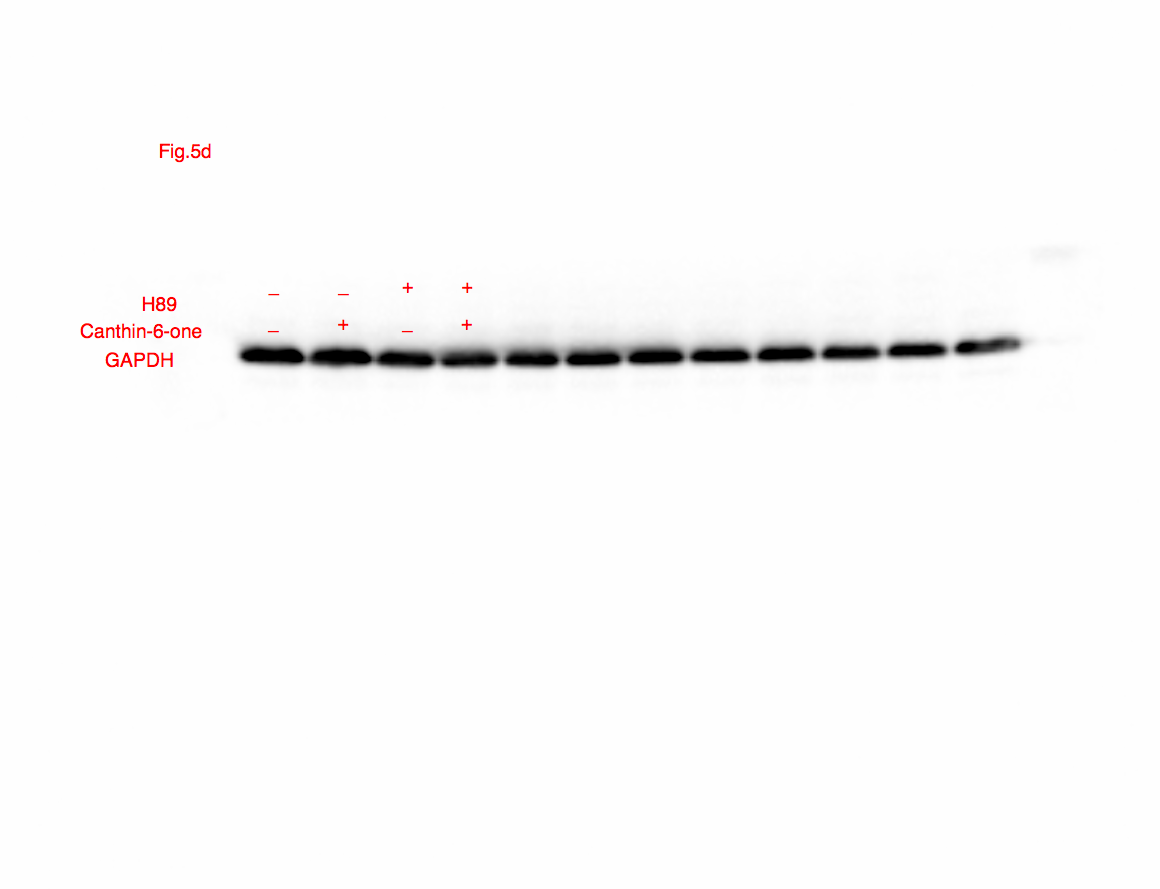

Supplement: DATA SHEET S1 — All western blot raw data. [file Data_Sheet_1.ZIP › wb origin/fig.5/d/GAPDH.tif]

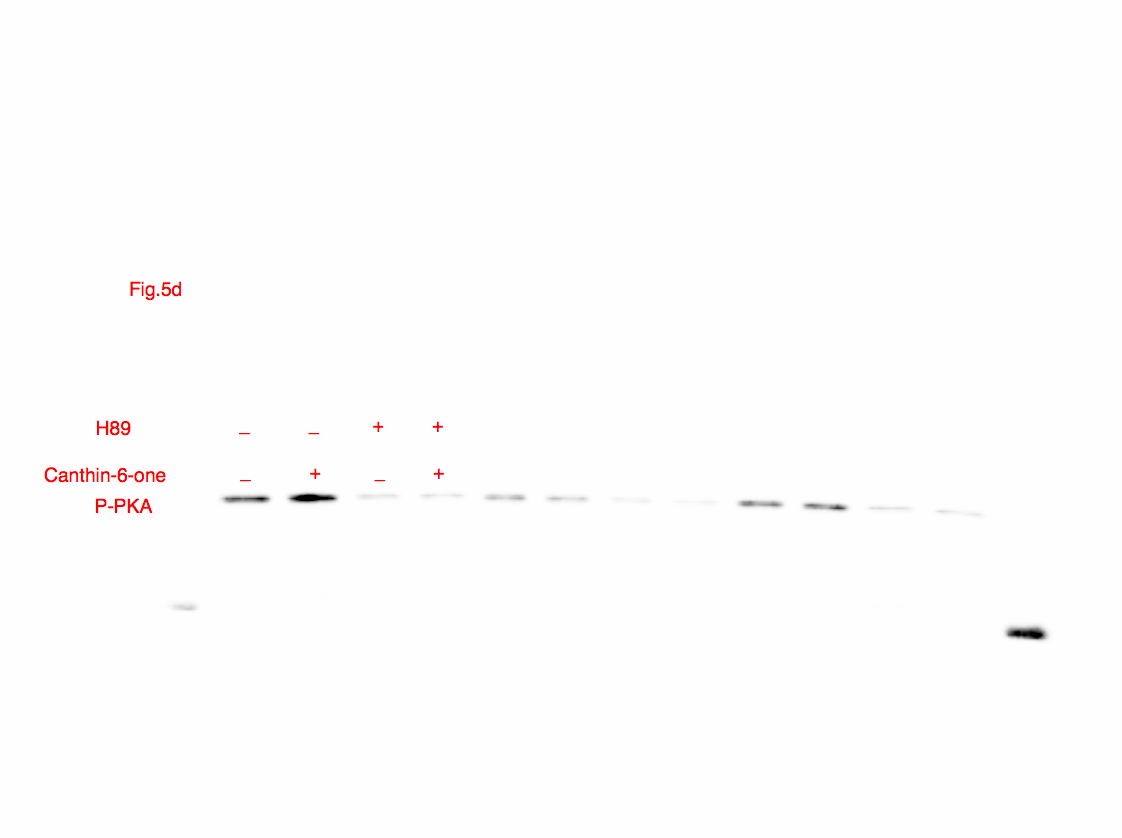

Supplement: DATA SHEET S1 — All western blot raw data. [file Data_Sheet_1.ZIP › wb origin/fig.5/d/p-PKA.tif]

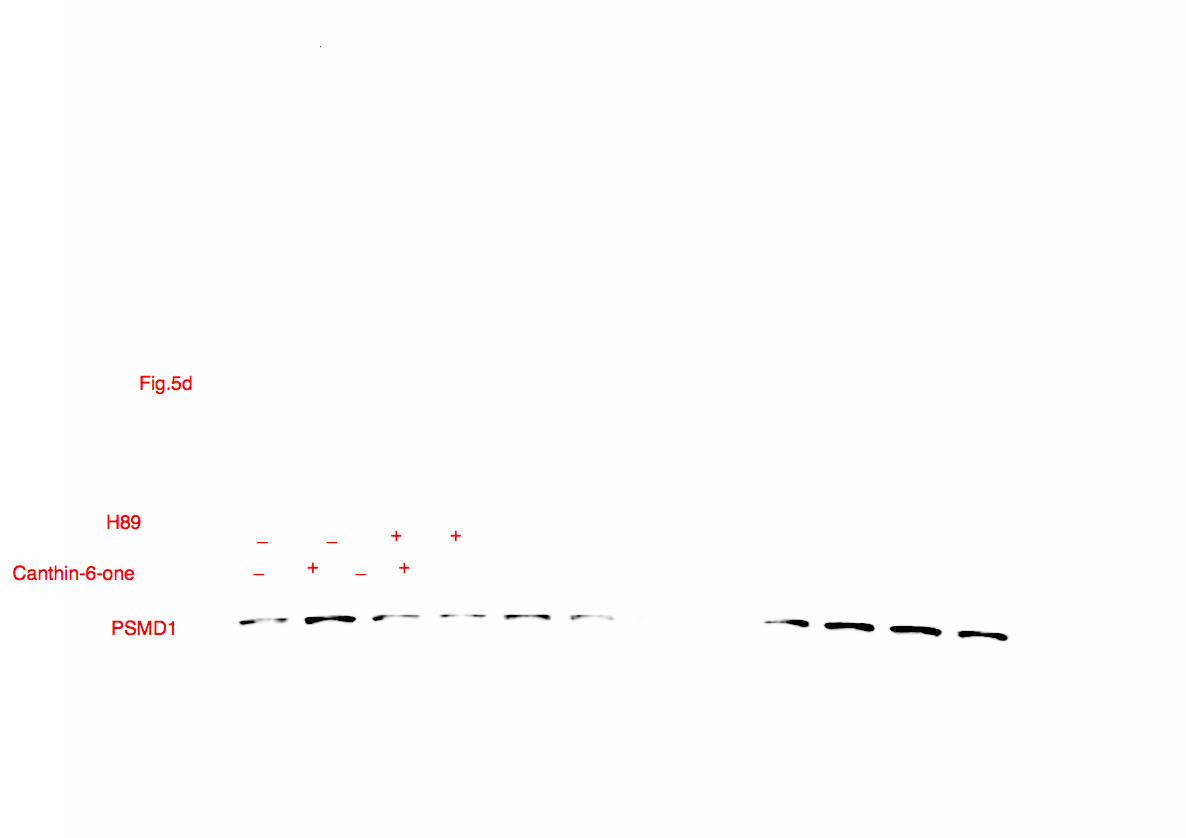

Supplement: DATA SHEET S1 — All western blot raw data. [file Data_Sheet_1.ZIP › wb origin/fig.5/d/PSMD1.tif]

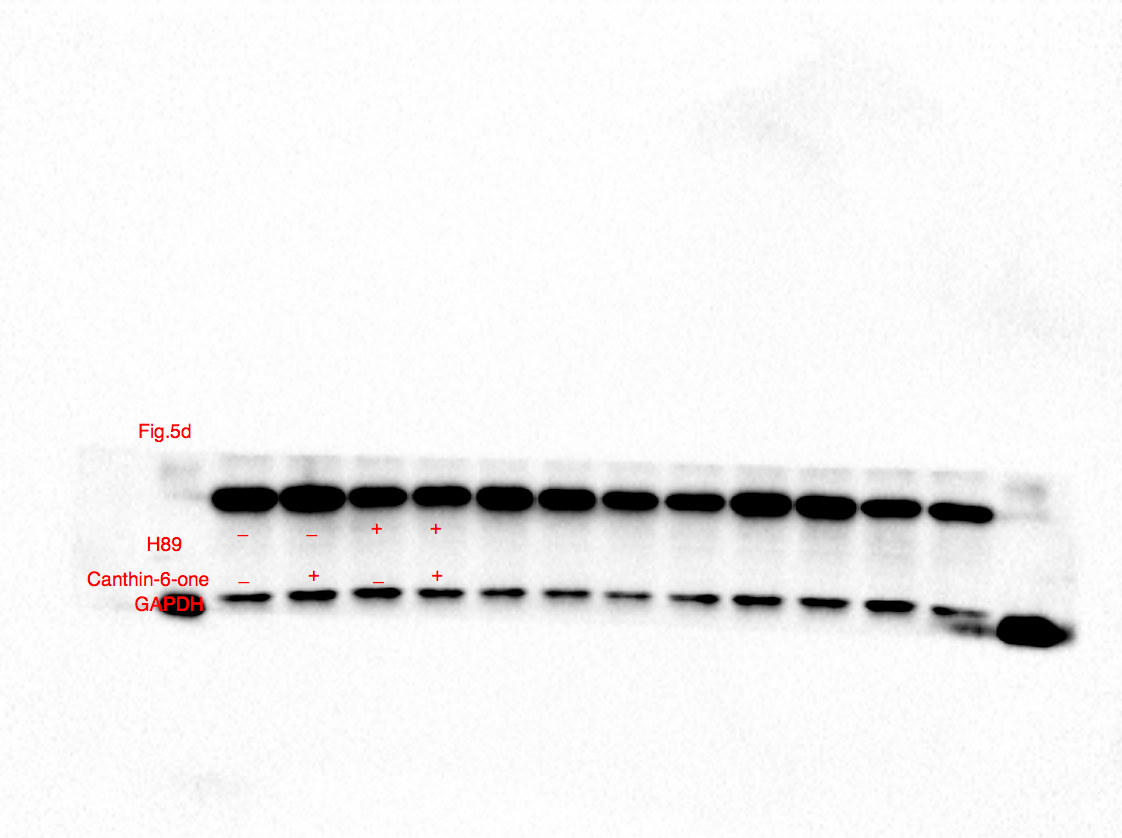

Supplement: DATA SHEET S1 — All western blot raw data. [file Data_Sheet_1.ZIP › wb origin/fig.5/d/T-PKA..tif]

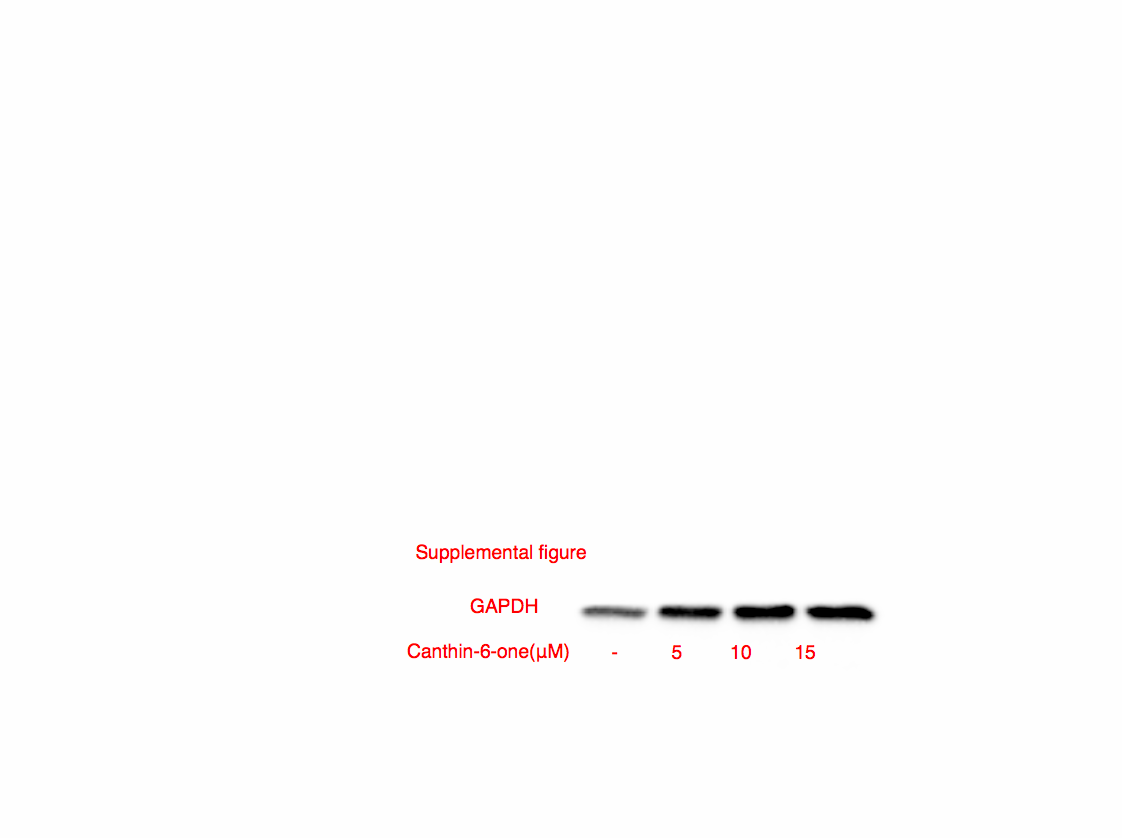

Supplement: DATA SHEET S1 — All western blot raw data. [file Data_Sheet_1.ZIP › wb origin/TAU/gapdh.tif]

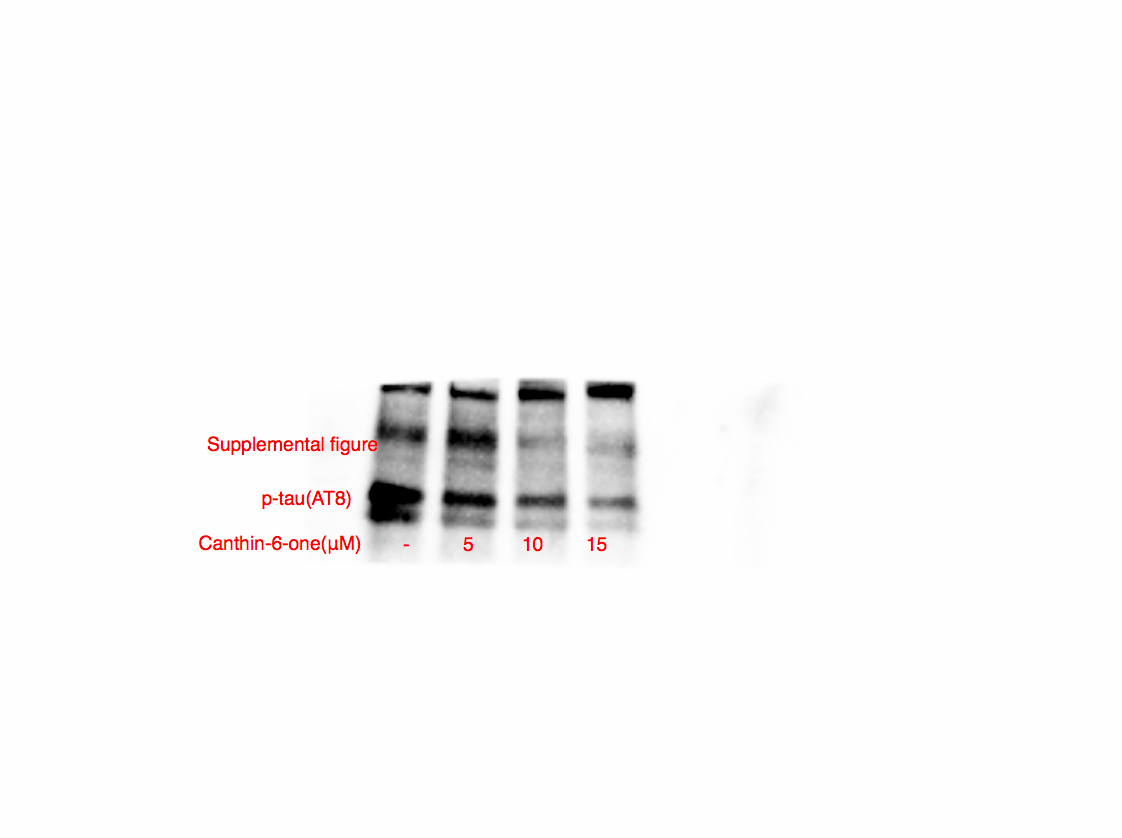

Supplement: DATA SHEET S1 — All western blot raw data. [file Data_Sheet_1.ZIP › wb origin/TAU/T-tau1.tif]
